# Optical resolution *via* chiral auxiliaries of curved subphthalocyanine aromatics†

**DOI:** 10.1039/d4sc06241h

**Published:** 2024-10-28

**Authors:** Giulia Lavarda, Lara Tejerina, Tomás Torres, M. Victoria Martínez-Díaz

**Affiliations:** a Department of Organic Chemistry, Universidad Autónoma de Madrid Madrid 28049 Spain victoria.martinez@uam.es tomas.torres@uam.es; b Institute for Advanced Research in Chemical Sciences, Universidad Autónoma de Madrid Madrid 28049 Spain; c IMDEA-Nanociencia c/Faraday, 9, Cantoblanco Madrid 28049 Spain

## Abstract

Chiral conjugated materials with curved topologies hold significant promise for advanced optoelectronic applications. Among these, bowl-shaped subphthalocyanine (SubPc) aromatics are particularly noteworthy due to their superb optoelectronic properties and synthetic versatility. Despite their potential, the development and application of inherently chiral SubPcs as functional materials have been hampered by the scalability and feasibility limitations of current high-performance liquid chromatography methods. In this work, we employ axial derivatization with BINOL-based chiral auxiliaries to achieve the optical resolution of *C*_3_-symmetric SubPcs. This approach allows us to obtain optically active *meta* and *ortho*-substituted SubPc derivatives in high yields and enantiomeric excess through straightforward organic chemistry protocols. In addition, we serendipitously observe unprecedented bowl-to-bowl inversion of the SubPc macrocycle upon removal of the derivatizing ligand under specific experimental conditions. These findings represent a significant milestone in the study of chirality in curved aromatics.

## Abstract 2

We report the optical resolution of inherently chiral, bowl-shaped subphthalocyanines (SubPcs) using BINOL-based chiral auxiliaries. Additionally, unprecedented bowl-to-bowl inversion of the SubPc macrocycle is observed under specific conditions.

## Graphical abstract

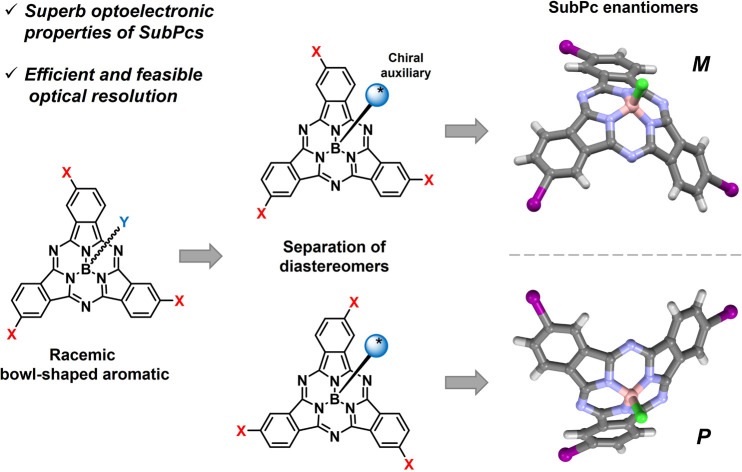

## Introduction

Inspired by the homochiral nature of life, the study of chirality is a major focus in chemical research.^1^ While its critical role in biomedical applications is well established, this symmetry property has only recently emerged as a design principle for advanced functional materials in organic optoelectronics.^2^ In this context, the implementation of chiral elements endows molecular systems with unique properties such as chiroptical responses^3–5^ and chiral-induced spin selectivity.^6,7^ In addition, chirality-dependent organization allows for unprecedented control over conductivity and exciton diffusion by simply tuning the optical purity of optoelectronic materials.^2,4,8–12^

**Fig. 1:**
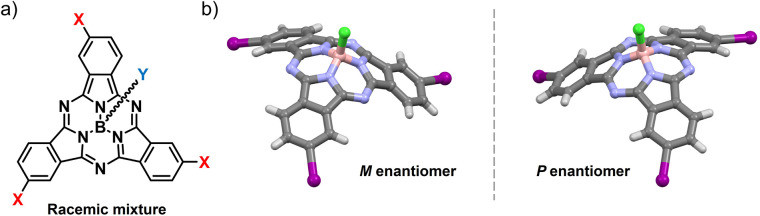
(a) Molecular structure of a racemic *meta*-substituted, *C*_3_-symmetric SubPc. (b) *M* and *P* enantiomers of *meta*-substituted, *C*_3_-symmetric I_3_SubPc-Cl.

Conjugated molecules – systems of choice for technological applications due to their mechanical, optical and electronic properties – pose a challenge in achieving inherently chiral structures.^2,4,13,14^ Introducing curvature into π-systems is an elegant strategy to expand the chemical space for chiral photo- and electroactive materials.^15–19^ Among the few known examples of bowl-shaped aromatics, subphthalocyanines (SubPcs) stand out for their superb optoelectronic properties and synthetic versatility.^20,21^ Their 14π-electron aromatic core and non-planar geometry – conferred by the tetrahedral coordination of the central boron atom – result in robust light-harvesting features, intense fluorescence emission, and excellent charge transport properties, the latter resulting from a permanent dipole moment and the ability to organize into ordered columnar assemblies.^12,21–26^ By virtue of these unique features, SubPcs have emerged as high-performing molecular materials in many fields of application, ranging from photovoltaics to emission technologies.^21,24^ Moreover, the cone-shaped geometry of these contracted porphyrinoids renders them intrinsically non-centrosymmetric. Thus, inherently chiral SubPcs are obtained from cyclotrimerization of non-*C*_2v_ phthalonitrile precursors (*e.g.*, *meta*- or *ortho*-monosubstituted dicyanobenzenes) as racemic mixtures of enantiomers (namely, *P* and *M* for the *C*_3_ regioisomer, Fig. 1).^27^

Due to their unique structural, optical and electronic properties, optically pure SubPcs are highly attractive value-added materials for advanced applications.^12,28^ Since the first report on the analytical resolution of racemic SubPcs by chiral high-performance liquid chromatography (HPLC),^29^ most efforts to obtain enantiopure SubPcs have focused on optimizing the scalability of the HPLC methodology.^12,21,30,31^ However, the intrinsic limitations of the HPLC technique (low throughput, cost, time consumption due to the need for multiple runs and method development), together with specific solubility issues of the SubPc framework, hamper scalability and feasibility, thus hindering the exploitation of curved SubPc aromatics as chiral materials. On the other hand, the exploration of alternative procedures (namely, optical resolution with chiral auxiliaries or *de novo* asymmetric synthesis^32,33^) for the preparation of enantiopure SubPcs by common organic laboratory techniques remains a pending task. Indeed, the former approach has received little attention in the literature in the frame of chiral buckybowls and contracted porphyrinoid aromatics.

Here, we report the efficient optical resolution of inherently chiral *C*_3_-symmetric SubPcs *via* axial derivatization with BINOL-based chiral auxiliaries. Moreover, unprecedented evidence for bowl-to-bowl inversion of the SubPc framework is serendipitously observed upon removal of the derivatizing ligand under specific conditions. Besides their interest as functional materials, enantiopure SubPcs thus also emerge as a precious tool for the in-depth study of SubPc reactivity, which mechanistic aspects have only rarely been addressed.^21,34–36^

## Results and discussion

### Chiral SubPc platforms and derivatization strategy

*C*_3_-symmetric, *meta*- and *ortho*-substituted I_3_SubPc-Cl *m*-1 and *o*-1 were selected as model chiral SubPc derivatives. The halogen atom at the axial position of the macrocycle is known to play a key role in imparting directional columnar packing of the SubPc framework, which is crucial for charge- and exciton transport applications.^12,22,23^ In addition, I_3_SubPc-Cl precursors serve as ideal platforms for the preparation of targeted SubPc-based systems through post-functionalization of the axial and peripheral positions *via* straightforward methodologies, making their optical resolution of strategic importance.^21^ On the other hand, axially chiral BINOL derivatives were exploited as resolving agents.^37,38^ Due to the high configurational stability of the enantiopure atropoisomers, binaphthyls are compounds of choice in asymmetric synthesis and catalysis.^39,40^ As a derivatization strategy, axial ligand exchange was preferred over peripheral functionalization, as both the replacement of the apical halogen and the subsequent removal of the resolving agent can be achieved *via* simple chemical protocols.^21,41–43^

SubPcs *m*-1 and *o*-1 were prepared as racemic mixtures by cyclotrimerization of 4-iodophthalonitrile and 3-iodophthalonitrile, respectively, in the presence of BCl_3_ (see ESI, Section 4† for the analysis of inherent chirality of *C*_3_-symmetric SubPcs).^12,44^

### Optical resolution of *meta*-substituted SubPc

The optical resolution of *m*-1 was first attempted by replacing the chlorine ligand with (*R*)-BINOL as a chiral auxiliary after prior activation of the SubPc axial position with AlCl_3_ (Scheme S1†).^42^ This approach allows for milder reaction conditions compared to one-step axial substitution reactions, thus preserving the optical purity of the resolving agent.^45^ The formation of I_3_SubPc-BINOL *m*-2 (46% yield) as a 1 : 1 mixture of diastereomers was evidenced by the presence of two distinct signal sets in the corresponding ^1^H NMR spectrum and their integral ratio (Fig. S1†).^46^ However, separation of the diastereomers by column chromatography on silica gel proved infeasible (further details in ESI, Section 2†).

**Fig. 2:**
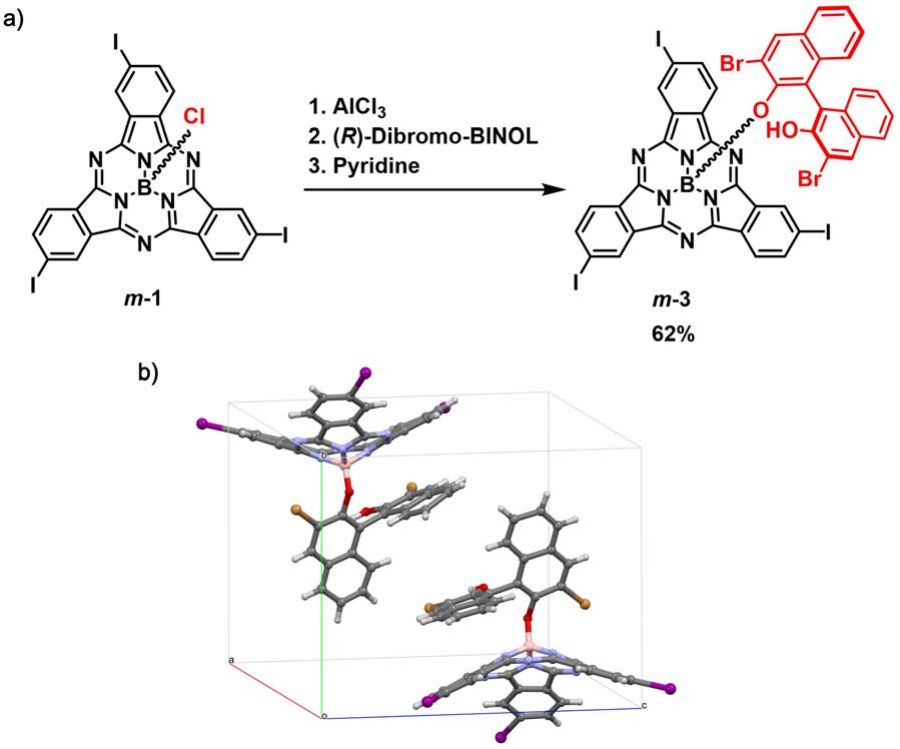
(a) Synthetic scheme for the preparation of *m*-3 as a mixture of diastereomers from racemic *m*-1*via* activation of the axial position with AlCl_3_. (b) Asymmetric unit of the crystal structure of *m*-3 showing the presence of two diastereomeric species. Toluene molecule of crystallization has been omitted for clarity.

**Fig. 3:**
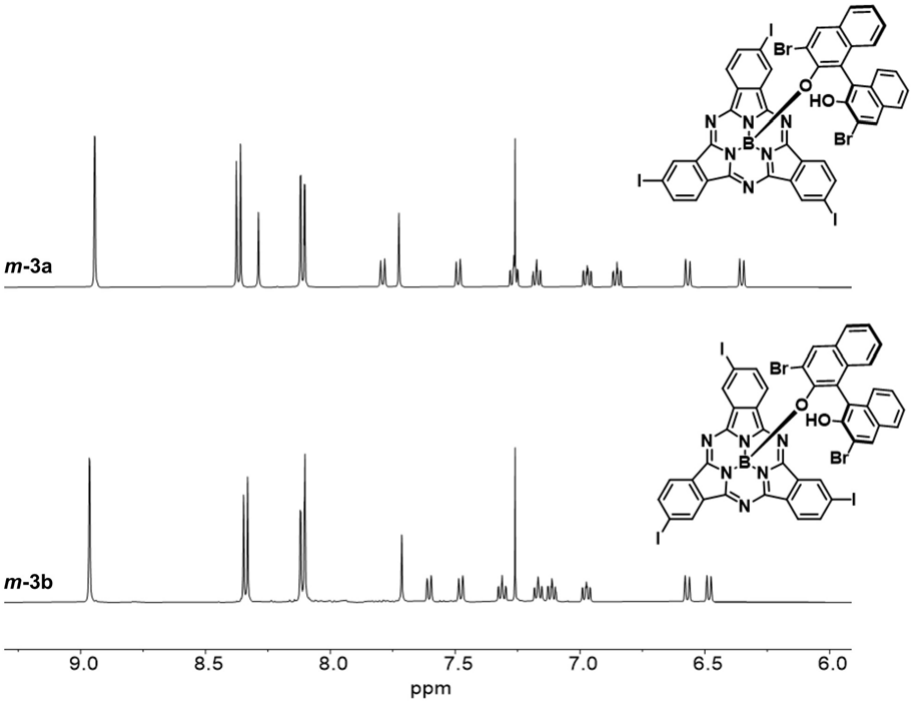
^1^H NMR spectra (500 MHz, chloroform-*d*_1_) of *m*-3a and *m*-3b diastereomers.

On the other hand, derivatization of *m*-1 with (*R*)-(+)-3,3′-dibromo-1,1′-bi-2-naphthol as chiral auxiliary afforded *m*-3 (62% yield, Fig. 2a and Scheme S2†), which showed two distinct spots on thin layer chromatography (*R*_f_ = 0.36 and 0.31 in dichloromethane/heptane 3 : 1).^47^ As observed for *m*-2, the ^1^H NMR spectrum of *m*-3 confirmed the formation of a 1 : 1 mixture of diastereomers (Fig. S7†). Accordingly, the HPLC chromatogram of the diastereomeric mixture revealed two distinct peaks with similar relative areas (Fig. S41†). The two diastereomers can be distinguished within the asymmetric unit in the single crystal structure of *m*-3, as evidenced by X-ray diffraction analysis (Fig. 2b and Tables S3 and S4†).^48^ Separation of the two diastereomers was achieved by column chromatography on silica gel yielding *m*-3a (first eluted diastereomer) and *m*-3b (second eluted diastereomer) in 28% and 27% yield, respectively (Fig. S6†).^49^ While *m*-3a was obtained as 97.2% pure, the second collected fraction (enriched in *m*-3b) appeared to contain 10.6% *m*-3a, as determined by HPLC (Fig. S42 and S43†). Further purification of the second eluted fraction through a second chromatographic column yielded *m*-3b as a pure fraction (Fig. S44†). The NMR spectra of the isolated *m*-3a and *m*-3b diastereomers are shown in Fig. 3 and S8–S18.†

**Fig. 4:**
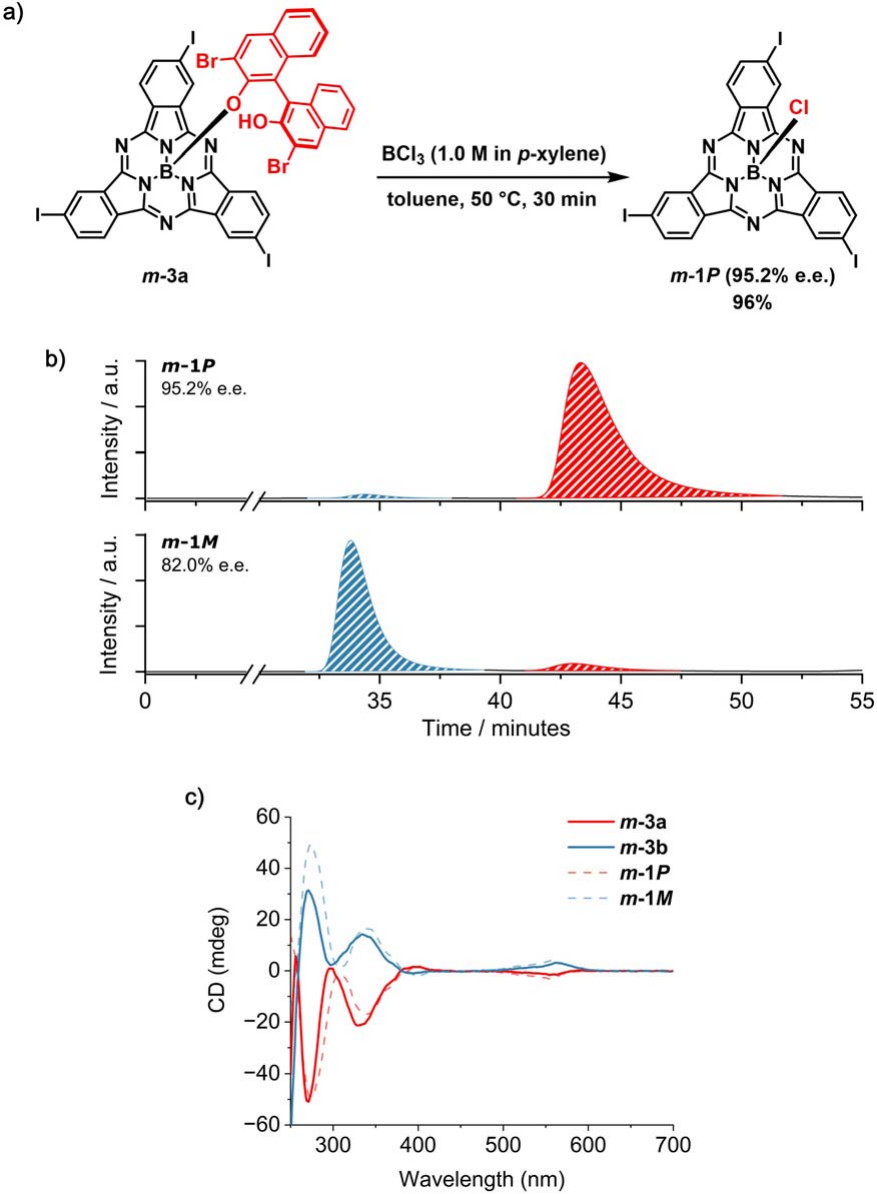
(a) Synthetic scheme for the removal of the BINOL-based chiral auxiliary from *m*-3a in the presence of BCl_3_ to afford *m*-1*P* (95.2% e.e.). (b) HPLC chromatograms of the products obtained from the removal of the chiral auxiliary from *m*-3a (top) and *m*-3b (bottom). The blue trace indicates the peak corresponding to the *M* enantiomer, whereas the red trace indicates the peak corresponding to the *P* enantiomer. Eluting solvent: toluene/*n*-hexane 50 : 50 (*v*/*v*); flow rate = 1.2 mL min^−1^; temperature = 10 °C; detection wavelength = 570 nm). (c) CD spectra of *m*-3a (red spectrum, full line), *m*-3b (blue spectrum, full line), *m*-1*P* (red spectrum, dotted line) and *m*-1*M* (blue spectrum, dotted line) in CHCl_3_ (2.0 × 10^−5^ M).

At this point, the resolving agent was removed by treating the separated *m*-3a and *m*-3b species with an excess of BCl_3_ (10 equiv) at 50 °C in toluene (Fig. 4a and Schemes S5 and S6†). Under these conditions, *m*-1*P* and *m*-1*M* were obtained from *m*-3a and *m*-3b, respectively, in excellent yield (namely 96%) and high enantiomeric excess (e.e. 95.2% and 82.0%, Fig. 4b, S45 and S46†). The slightly lower purity of *m*-1*M* is consistent with that of its diastereomeric precursor *m*-3b, obtained as the second eluted fraction from the first column chromatography (*vide supra*). Comparison of the retention time of the I_3_SubPc-Cl enantiomers obtained by removal of the chiral auxiliary from *m*-3a and *m*-3b (Fig. 4b) with those of the *m*-1 enantiomers resolved by chiral HPLC,^12^ allows assignment of the absolute configuration of the separated species. The enantiomeric relationship between the *m*-1*P* and *m*-1*M* fractions obtained by axial ligand exchange from *m*-3a and *m*-3b was further confirmed by CD spectroscopy (Fig. 4c). From a qualitative comparison of the CD profiles of the isolated diastereomers (*m*-3a and *m*-3b) and the resulting enantiomers (*m*-1*P* and *m*-1*M*), and taking into account the similar purity of the diastereomeric precursors and enantiomeric products, we infer that the absolute configuration is retained upon removal of the chiral auxiliary under the conditions investigated.^50^ Thus, the exchange of the axial binaphthoxy ligand is likely to occur by interaction of the boron halide with the central boron atom of the SubPc framework from the convex face of the cone-shaped macrocycle. This is consistent with the bimolecular σ-bond metathesis mechanism postulated for axial ligand exchange between SubPc-Cl derivatives and phenols.^35^

### Optical resolution of *ortho*-substituted SubPc

Different results were obtained for the *ortho*-substituted SubPc analog *o*-1. In this case, axial derivatization of the racemic I_3_SubPc-Cl precursor with (*R*)-BINOL *via* AlCl_3_ activation led to the formation of *o*-2 (52% overall yield) as a mixture of diastereomers (*o*-2a and *o*-2b) which were readily separated by column chromatography on silica gel (*R*_f_*o*-2a = 0.38 and *R*_f_*o*-2b = 0.30 in toluene) in 24% yield each (Scheme S3 and Fig. S23–S32†).^51^ At this point, removal of the chiral auxiliary from the isolated diastereomers was expected to yield the enantiomerically pure *P* and *M* I_3_SubPc-Cl derivatives, as observed for *meta*-substituted *m*-3. However, treatment of *o*-2b with 20 equiv. BCl_3_ at 70 °C for 1 hour afforded *o*-1b with an e.e. of 68.2% (Scheme S7 and Fig. S47†). This low e.e., compared to the purity of the diastereomeric precursor,^52^ indicates that a partial bowl-to-bowl inversion of the macrocycle occurs concomitantly with the axial nucleophilic substitution under the conditions investigated.^53,54^ These findings provide the first experimental evidence for bowl-to-bowl inversion of SubPc derivatives in solution.^55–57^

**Fig. 5:**
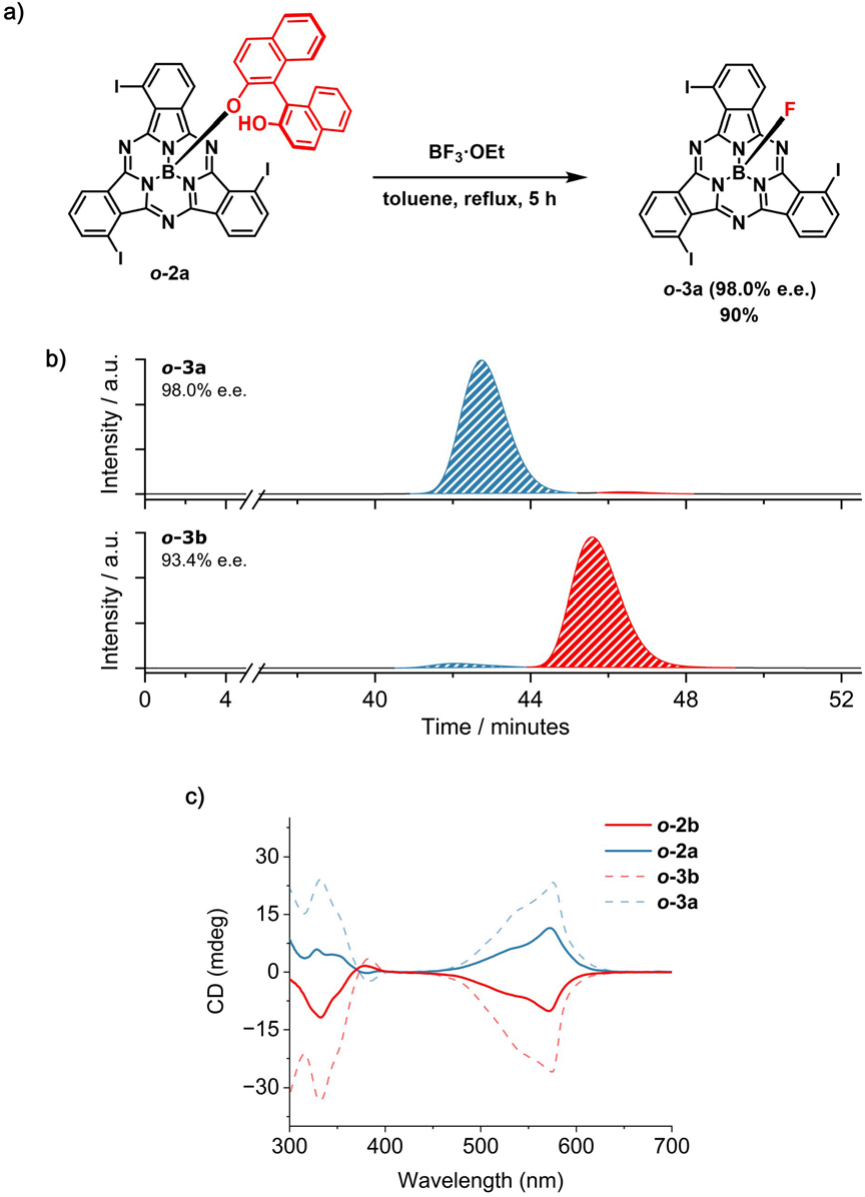
(a) Synthetic scheme for the removal of the BINOL chiral auxiliary from *o*-2a in the presence of BF_3_·Et_2_O to afford *o*-3a (98.0% e.e.). The absolute configuration of the SubPc macrocycle in the starting material and in the final product is not known.^59^ For visualization purposes, the *M* configuration has been depicted. (b) HPLC chromatograms of the products obtained from the removal of the chiral auxiliary from *o*-2a (top) and *o*-2b (bottom) in the presence of BF_3_·Et_2_O. Eluting solvent: toluene/*n*-hexane 80 : 20 (*v*/*v*); flow rate = 0.6 mL min^−1^; temperature = 20 °C; detection wavelength = 580 nm. (c) CD spectra of *o*-2a (blue spectrum, full line), *o*-2b (red spectrum, full line), *o*-3a (blue spectrum, dotted line) and *o*-3b (red spectrum, dotted line) in CHCl_3_ (5.0 × 10^−5^ M).

At this point, the configurational stability of the *ortho*-substituted derivatives was tested in the presence of BF_3_·Et_2_O as a Lewis acid. Interestingly, by removing the axial BINOL ligand with an excess of BF_3_·Et_2_O (25 equiv) in refluxing toluene, *o*-3a and *o*-3b were obtained in 90% yield and e.e. of 98.0% and 93.4%, respectively (Schemes S8, S9, Fig. 5a, b, S33–S38, S51 and S52†).^58,59^ As observed for *m*-1*P* and *m*-1*M*, the CD spectra of *o*-3a and *o*-3b are mirror images, with the signs of the bands remaining unchanged when diastereomeric (*i.e.*, *o*-2a and *o*-2b) and resulting enantiomeric fractions (*o*-3a and *o*-3b) are compared (Fig. 5c, S54 and S55†). This suggests that the axial nucleophilic substitution for the *ortho*-substituted *o*-2 in the presence of BF_3_·Et_2_O under the investigated conditions proceeds with retention of the configuration.

## Conclusions

In conclusion, we report herein the unprecedent optical resolution of inherently chiral bowl-shaped SubPc aromatics by derivatization with chiral auxiliaries, along with the first experimental evidence of bowl-to-bowl inversion of the SubPc macrocycle in solution. The chiral resolution of triiodo-substituted, *C*_3_-symmetric SubPc derivatives was successfully achieved by functionalization with enantiopure BINOL-based ligands upon activation of the apical position of the macrocycle, allowing for a facile separation of the resulting diastereomers by column chromatography on silica gel. Subsequent removal of the chiral auxiliary from the isolated diastereomeric fractions in the presence of boron halides afford optically active I_3_SubPc-X (X = Cl or F) in high yield (96%) and purity (97.7% and 99.0% for *m*-1*M* and *o*-3a, respectively). This approach, which has never been exploited for the optical resolution of contracted porphyrinoids, constitutes a feasible and efficient method for the preparation of enantiopure SubPcs using simple organic chemistry protocols. Remarkably, experimental evidence of bowl inversion of the SubPc framework was serendipitously observed in the axial ligand exchange on the *ortho*-substituted derivative in the presence of BCl_3_ as a Lewis acid. These path-breaking results open the door for in-depth mechanistic studies of SubPc reactivity and pave the way for the exploitation of enantiopure SubPcs as functional materials.

## Data availability

All experimental procedures, and compound characterization data are provided in the ESI.† Additional datasets and materials related to this study are available from the corresponding authors upon reasonable request.

## Author contributions

M. V. M.-D. and T. T. designed and supervised the research. G. L. carried out the experiments related to the *meta*-substituted SubPcs. L. T. performed the experimental work related to the *ortho*-substituted SubPcs. G. L. wrote the manuscript. M. V. M.-D. and T. T. corrected it. Overall, G. L. and L. T. contributed equally to this work.

## Conflicts of interest

There are no conflicts to declare.

## Supplementary Material

SC-OLF-D4SC06241H-s001
