# Supplementary material for: Optical resolution via chiral auxiliaries of curved subphthalocyanine aromatics
Source: Chem Sci. 2024 Oct 28;15(46):19369–74. doi: 10.1039/d4sc06241h (PMC11575623; doi:10.1039/d4sc06241h)
Supplement: SC-015-D4SC06241H-s001 [file SC-015-D4SC06241H-s001.pdf]

Supplementary Information (SI) for Chemical Science.
This journal is © The Royal Society of Chemistry 2024

Supporting Information

Optical resolution *via* chiral auxiliaries of curved subphthalocyanine aromatics

Giulia Lavarda,†[a] *Lara Tejerina,*†[a]
*Tomás Torres**[a,b,c], *and M. Victoria Martínez-Díaz**[a,b]

[a] Department of Organic Chemistry, Universidad Autónoma de Madrid, 28049 Madrid, Spain.

[b] Institute for Advanced Research in Chemical Sciences, Universidad Autónoma de Madrid, 28049 Madrid, Spain.

[c] IMDEA-Nanociencia, c/Faraday, 9, Cantoblanco, 28049 Madrid, Spain.

† These authors contributed equally to this work.

* Correspondence to: victoria.martinez@uam.es, tomas.torres@uam.es

Table of Contents

1. Materials and methods……………………………………………………………………………………S1

2. Synthesis and characterization of BINOL-functionalized SubPcs ***m*-2**, ***m*-3** and ***o*-2**….S2

3. Synthetic procedures for the removal of the chiral auxiliary from ***m*-3** and ***o*-2**………S26

4. Analysis of inherent chirality of C_3-symmetric SubPcs……………………………………S32

5. Additional experimental data……………………………………………………………………S34

5.1 X-ray diffraction analysis…………………………………………………………………S34

5.2 HPLC chromatograms……………………………………………………………………S38

5.3 Circular dichroism and UV-vis absorption spectra ……………………………………S44

6. Author contributions……………………………………………………………………………S45

7. References………………………………………………………………………………………S46

1. Materials and methods

Chemicals (reagent grade) and solvents (synthetic grade, anhydrous, HPLC grade, spectroscopic grade and deuterated) were purchased from Aldrich Chemical, Alfa Aesar, Acros Organics, TCI and Scharlau and used as received. I_3SubPc-Cl derivatives ***m*-1**[1] and ***o*-1**[2] were prepared following previously reported synthetic procedures and obtained as racemic mixtures of *M* and *P* enantiomers (see section 4 for the analysis of inherent chirality of C_3-symmetric SubPcs). Thin layer chromatography (TLC) was carried out on aluminum sheets precoated with silica gel 60 F254 0.2 mm (Merck). Column chromatography was performed using silica gel (230-400 mesh, 0.040-0.063 mm, Merck). High-performance liquid chromatography (HPLC) was performed on an Agilent 1200 instrument equipped with a Daicel CHIRALPAK IC semi-preparative chiral column (particle size: 5 µm; internal diameter: 10 mm; length: 250 mm; immobilized polysaccharide phase).

^{1}H and ^{13}C nuclear magnetic resonance (NMR) spectra were recorded on a Bruker AC-300 (300 MHz) or a Bruker DRX-500 (500 MHz) spectrometer at room temperature. Chemical shifts (δ) are expressed in ppm and referenced to the residual peak of the solvent. Fourier-transform infrared spectroscopy (FT-IR) was performed with a Bruker IFS66v FTIR spectrometer. UV-Vis absorption spectra were recorded employing a JASCO-V660 spectrophotometer. Circular dichroism (CD) spectroscopy was performed on a JASCO J-815 spectrometer equipped with a JASCO ETCT-72 temperature controller.

Matrix-assisted laser desorption/ionization time of flight (MALDI-TOF) mass spectrometry (MS) was performed with a Bruker Ultraflex III spectrometer equipped with a Nd:YAG laser operating at 355 nm. High resolution mass spectrometry (HR-MS) was measured employing ESI Positive Q-TOF using a Bruker Maxis II. (*trans*-2-[3-(4-tert-Butylphenyl)-2-methyl-2propenylidene]malononitrile (DCTB) was used as a matrix.

X-Ray diffraction analysis was with a Bruker KAPPA APEX II (X8 APEX) single-crystal diffractometer with Mo source (λ = 0.71073 Å). Data were collected utilizing a system equipped with an Oxford Cryosystems dispositive. Data are corrected with the SADABS program. Intensities are calculated with the SAINT software. The structures are resolved and refined using the Bruker SHELXTL Software Package. Single crystals of ***o*-2*** and ***m*-3** suitable for X-ray analysis were obtained by slow evaporation of chloroform or toluene, respectively, from a solution of the corresponding SubPc derivative. The deposition numbers of the resolved crystal structures in the Cambridge Crystallographic Data Centre are the following: CCDC 2376453 (***o*-2***) and 2376454 (***m*-3**). These data can be obtained free of charge from The Cambridge Crystallographic Data Centre.

Optical rotations were measured at 25 °C on an Anton Paar Modular Compact Polarimeter (MCP 150) using a 10 cm cell at 589 nm (sodium lamp).

2. Synthesis and characterization of BINOL-functionalized SubPcs *m*-2, *m*-3 and *o*-2

Synthesis and characterization of *m*-2 (mixture of diastereomers)

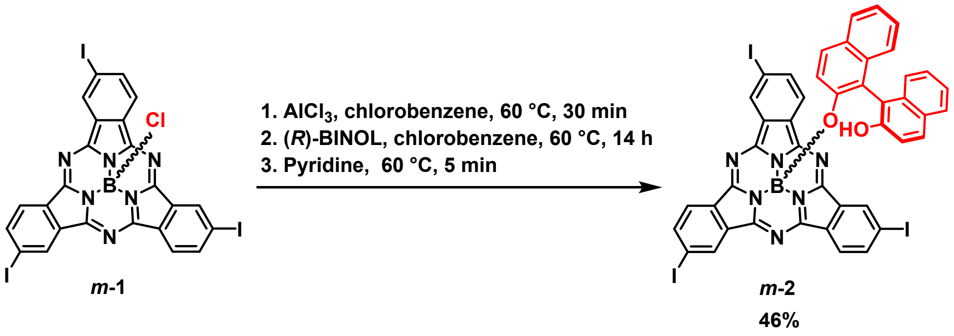

Scheme S1. Synthesis of ***m*-2** as a mixture of diastereomers from racemic ***m*-1** *via* activation of the axial position with $AlCl_3$.

I_3SubPc-Cl ***m*-1** (50.0 mg, 0.06 mmol) and $AlCl_3$ (12.4 mg, 0.09 mmol) were dissolved in anhydrous chlorobenzene (2 mL) under argon atmosphere in a 10 mL round bottom flask and the reaction mixture was stirred at 60 °C for 30 min. Then, a solution of (*R*)-(+)-1,1′-bi-2-naphthol (35.4 mg, 0.12 mmol) in anhydrous chlorobenzene (1 mL) was added, and the mixture was stirred at 60 °C for 14 h. At this point, pyridine (0.5 mL) was added, and the mixture was stirred for 5 min. The reaction mixture was then allowed to cool down to room temperature and the solvent was evaporated under reduced pressure. The crude product was purified as a mixture of diastereomers by column chromatography on silica gel using using dichloromethane/toluene 4:1 (*v*/*v*) as eluent. Recrystallization from a dichloromethane/methanol/water mixture afforded ***m*-2** as a purple solid in 46% yield (30 mg). Despite testing numerous eluent conditions in TLC, in no case two resolved spots were observed, and a column chromatography attempt carried out using the most promising eluent mixture among the ones tested on TLC (namely, $CHCl_3$/toluene 2:3) did not afford any pure diastereomeric fraction.

^{1}H NMR (500 MHz, chloroform-d_1): δ (ppm) = 8.97 (d, J = 1.5 Hz, 3H), 8.93 (dd, J_1 = 1.5 Hz, J_2 = 0.5 Hz, 3H), 8.34 (dd, J_1 = 8.0 Hz, J_2 = 0.5 Hz, 3H), 8.31 (dd, J_1 = 8.0 Hz, J_2 = 0.5 Hz, 3H), 8.12-8.09 (m, 6H), 7.91 (d, J = 8.5 Hz, 1H), 7.82 (d, J = 8.0 Hz, 1H), 7.79 (d, J = 8.5 Hz, 1H), 7.65-7.60 (m, 3H), 7.40 (d, J = 8.5 Hz, 1H), 7.37 (d, J = 8.5 Hz, 1H), 7.22-7.19 (m, 2H), 7.18-7.14 (m, 2H), 7.09-7.06 (m, 2H), 7.03-7.00 (m, 2H), 6.95-6.92 (m, 1H), 6.75-6.70 (m, 3H), 6.39 (d, J = 8.0 Hz, 1H), 6.29 (d, J = 8.5 Hz, 1H), 6.51-5.58 (m, 2H), 4.66 (s, broad), 2H).

^{13}C NMR (125.7 MHz, chloroform-d_1) : δ (ppm) = 151.09, 151.05, 150.97, 150.89, 149.91, 149.79, 147.98, 147.97, 138.60, 133.53, 133.52, 133.06, 133.04, 132.10, 132.03, 131.46, 131.40, 130.23, 130.21, 130.17, 129.97, 129.81, 129.73, 129.63, 128.87, 128.86, 127.90, 127.87, 127.84, 127.74, 127.74, 126.47, 126.36, 126.31, 126.29, 126.04, 125.26, 125.11, 124.59, 123.44, 123.41, 123.29, 120.75, 120.56, 118.56, 118.54, 114.79, 114.77, 96.12, 96.10.

^{11}B NMR (160.4 MHz, chloroform-d_1): δ (ppm) = -15.07.

MS (MALDI-TOF, DCTB): m/z = 1057.9 $[M]^+$.

HR-MS (MALDI-TOF, DCTB + PPG1000): m/z calculated for $C_{44}H_{22}BI_3N_6O_2$: 1057.9033; found: 1057.9030.

UV-vis (toluene): λ_{max} (nm) (log ε (dm^3 mol^{-1} cm^{-1})) = 574 (4.87), 556 (sh), 534 (sh), 326 (4.48), 272 (4.70).

FT-IR (ATR): ν (cm^{-1}) = 3521, 3056, 2919, 2853, 1694, 1598, 1551, 1503, 1439, 1385, 1261, 1233, 1169, 1141, 1077, 1056, 1042, 815, 765, 744, 703, 656, 598, 528.

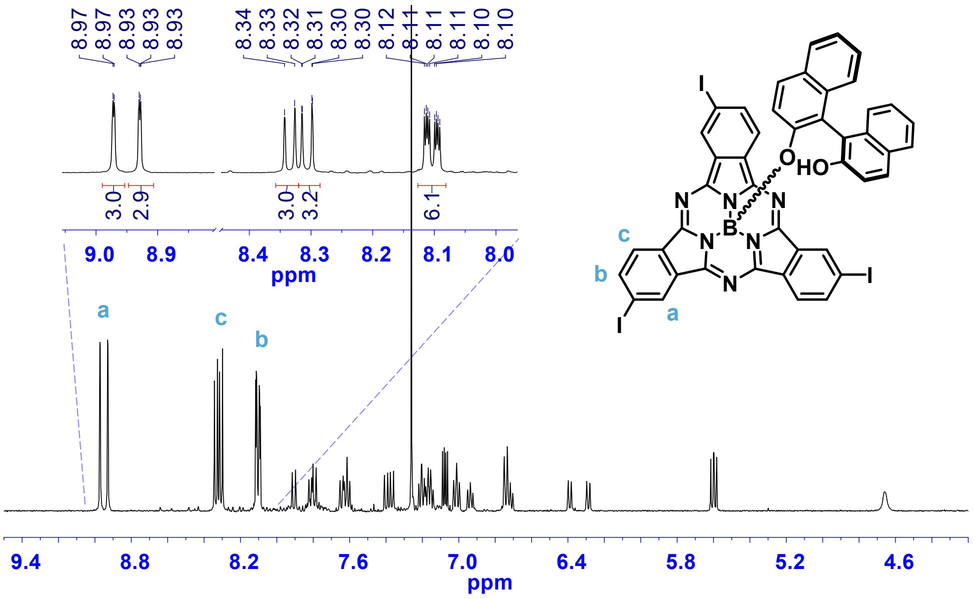

Figure S1. ^{1}H NMR spectrum (500 MHz, chloroform-d_1) of ***m*-2** (mixture of diastereomers). Inset: zoom of the signals of the isoindolic protons of the SubPc macrocycle.

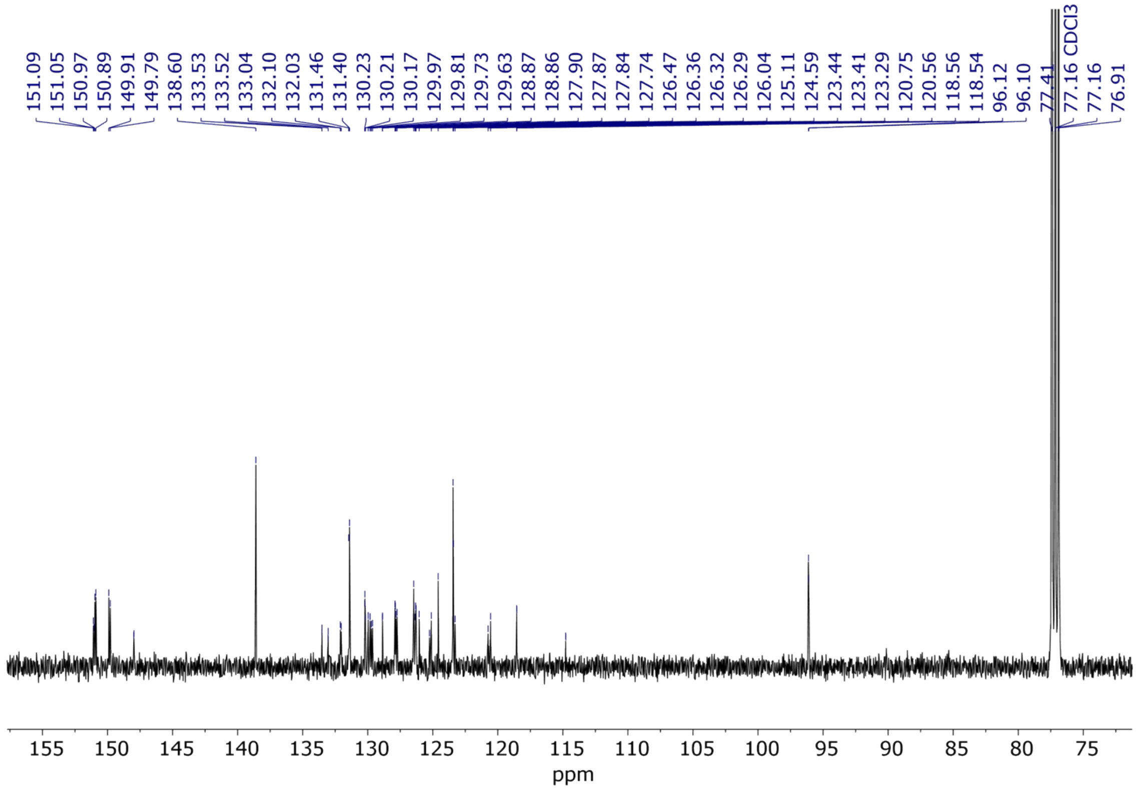

Figure S2. ^{13}C NMR spectrum (125.7 MHz, chloroform-d_1) of ***m*-2** (mixture of diastereomers).

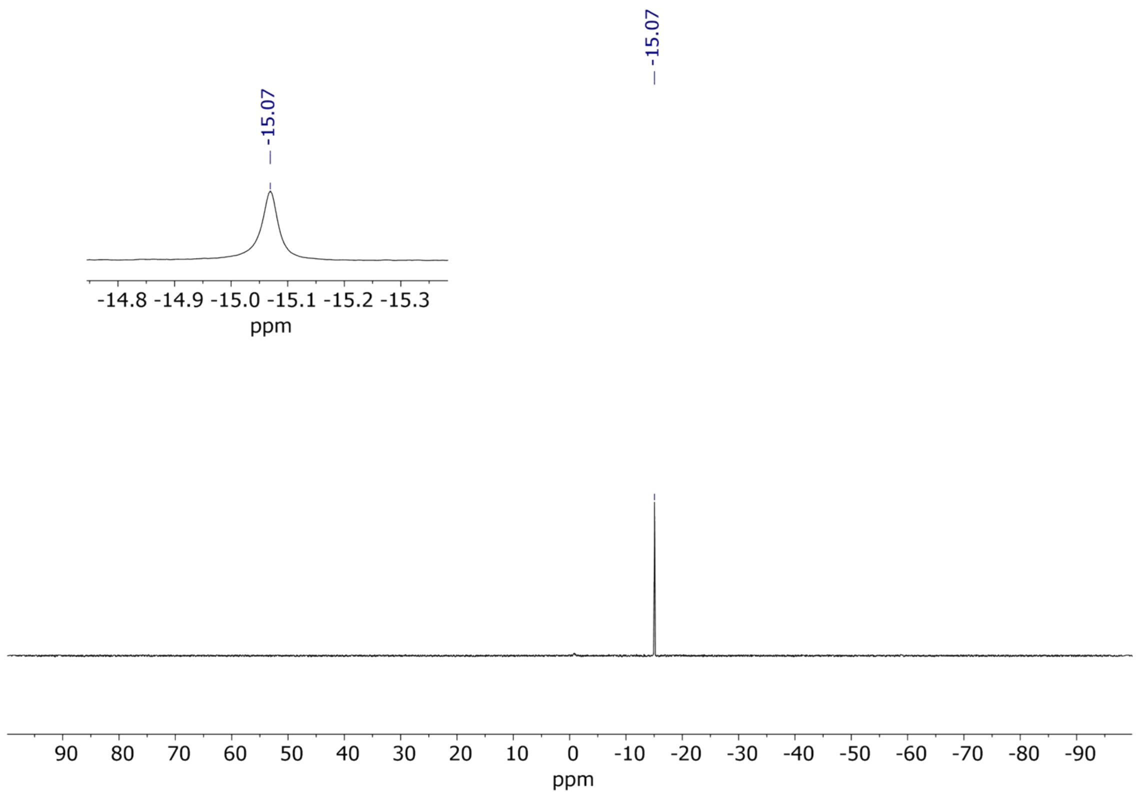

Figure S3. ^{11}B NMR spectrum (160.4 MHz, chloroform-d_1) of ***m*-2** (mixture of diastereomers).

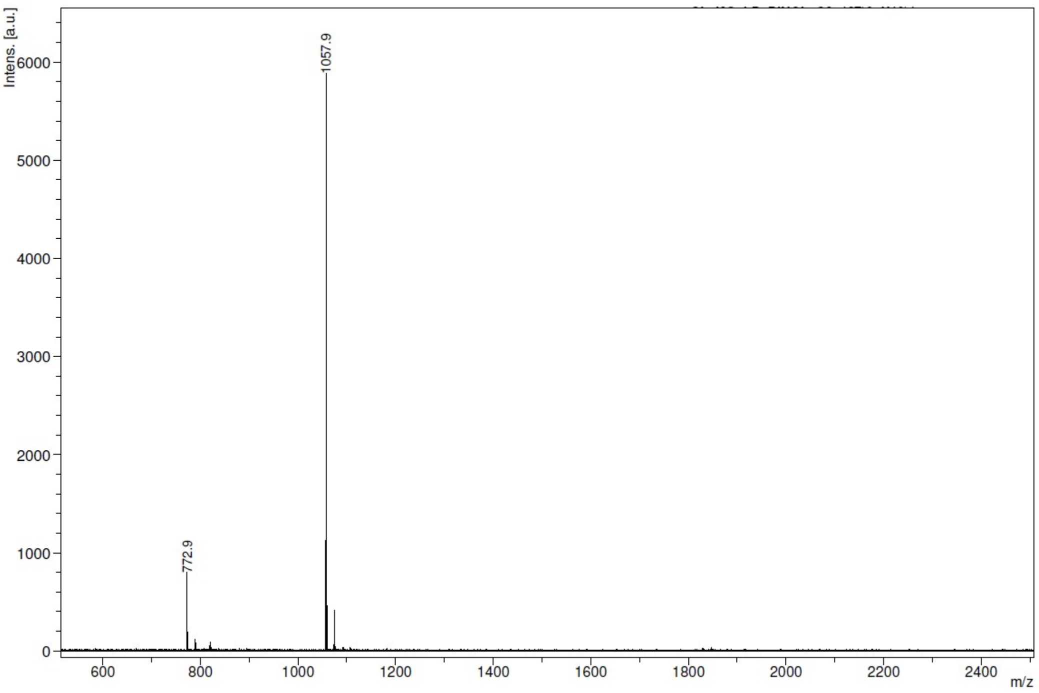

Figure S4. MALDI-TOF mass spectrum (matrix: DCTB) of ***m*-2** (mixture of diastereomers).

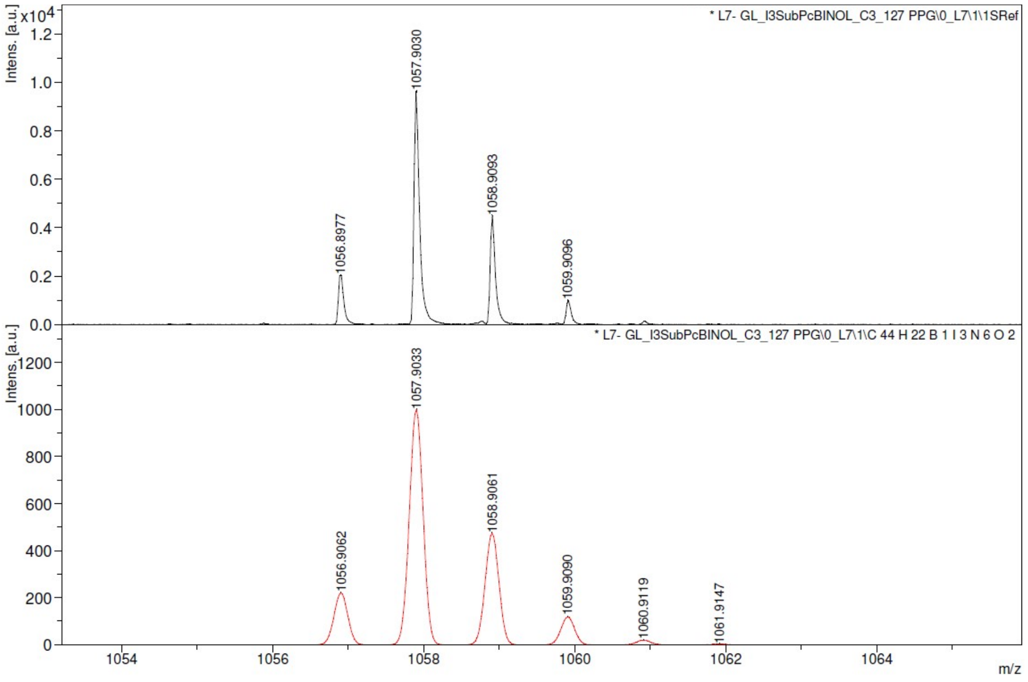

Figure S5. (Top) HR-MS MALDI-TOF spectrum (matrix: DCTB + PPG1000) and (bottom) calculated isotopic pattern of ***m*-2** (mixture of diastereomers).

Synthesis and characterization of *m*-3

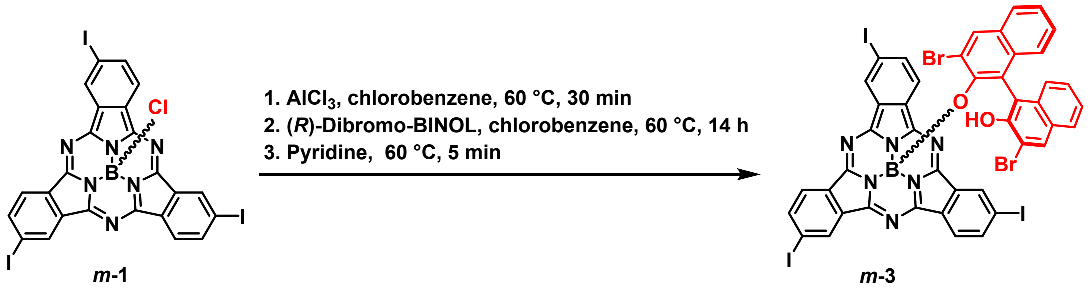

m-1 *m*-3 62%

Scheme S2. Synthesis of ***m*-3** as a mixture of diastereomers from racemic ***m*-1** *via* activation of the axial position with $AlCl_3$.

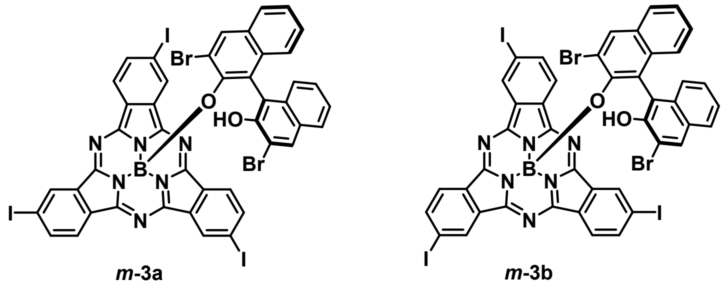

m-3a *m*-3b

Figure S6. Chemical structure of ***m*-3a** and ***m*-3b** diastereomers.

I_3SubPc-Cl ***m*-1** (50.0 mg, 0.06 mmol) and $AlCl_3$ (12.4 mg, 0.09 mmol) were dissolved in anhydrous chlorobenzene (2 mL) under argon atmosphere in a 10 mL round bottom flask and the reaction mixture was stirred at 60 °C for 30 min. Then, a solution of (*R*)-(+)-3,3′-dibromo-1,1′-bi-2-naphthol (27.5 mg, 0.06 mmol) in anhydrous chlorobenzene (2 mL) was added, and the mixture was stirred at 60 °C for 14 h. At this point, pyridine (0.5 mL) was added, and the mixture was stirred for 5 min. The reaction mixture was then allowed to cool down to room temperature and the solvent was evaporated under reduced pressure. The work-up procedures followed for the purification of ***m*-3** as a mixture of diastereomers and for the separation of ***m*-3a** and ***m*-3b** diastereomers are indicated below.

Purification of ***m*-3** *as a mixture of diastereomers.* The crude product was purified as a mixture of diastereomers by column chromatography on silica gel using dichloromethane/toluene 3:1 (*v*/*v*) as eluent. Recrystallization from a dichloromethane/methanol/water mixture afforded ***m*-3** as a purple solid in 62% yield (47 mg).

Purification of the isolated ***m*-3a** *and* ***m*-3b** *diastereomers.* The crude product was subjected to column chromatography on silica gel (6 cm ø × 23 cm) using dichloromethane/heptane 3:1 (*v*/*v*) as eluent. ***m*-3a** (first eluted diastereomer) and ***m*-3b** (second eluted diastereomer) were collected separately with a purity of 97.2% and 89.4%, respectively. The second eluted fraction was subjected to a second column chromatography on silica gel (6 cm ø × 23 cm, eluent: dichloromethane/heptane 3:1), which afforded pure ***m*-3b**. Recrystallization from a dichloromethane/MeOH/water

mixture afforded the isolated ***m*-3a** and ***m*-3b** diastereomers as purple solids in 28% and 27% yield, respectively.

m-3a *(first eluted diastereomer from column chromatography on silica gel)*

^{1}**H NMR** (500 MHz, chloroform-d_1): δ (ppm) = 8.74 (dd, J_1 = 1.5 Hz, J_2 = 0.5 Hz, 3H), 8.37 (dd, J_1 = 8.0 Hz, J_2 = 0.5 Hz, 3H), 8.29 (s, 1H), 8.11 (dd, J_1 = 8.0 Hz, J_2 = 1.5 Hz, 3H), 7.79 (d, J = 8.0 Hz, 1H), 7.73 (s, 1H), 7.49 (d, J = 8.0 Hz, 1H), 7.27 (ddd, J_1 = 8.0 Hz, J_2 = 6.8 Hz, J_3 = 1.0 Hz, 1H), 7.17 (ddd, J_1 = 8.0 Hz, J_2 = 6.8 Hz, J_3 = 1.0 Hz, 1H), 6.97 (ddd, J_1 = 8.5 Hz, J_2 = 6.8 Hz, J_3 = 1.0 Hz, 1H), 6.85 (ddd, J_1 = 8.5 Hz, J_2 = 6.8 Hz, J_3 = 1.0 Hz, 1H), 6.57 (d, J = 8.5 Hz, 1H), 6.35 (d, J = 8.5 Hz, 1H), 5.17 (s, 1H).

^{13}**C NMR** (125.7 MHz, chloroform-d_1): δ (ppm) = 151.18, 150.01, 147.38, 145.26, 138.61, 133.22, 132.94, 132.50, 132.09, 132.02, 131.40, 130.70, 129.69, 127.13, 126.99, 126.61, 126.59, 126.40, 125.76, 125.47, 124.90, 123.46, 122.84, 116.55, 116.41, 114.06, 96.12.

^{11}**B NMR** (160.4 MHz, chloroform-d_1): δ (ppm) = -14.92.

MS (MALDI-TOF, DCTB): m/z = 1215.7 $[M]^+$.

HR-MS (MALDI-TOF, DCTB + PPGNa 1000): m/z calculated for $C_{44}H_{20}BBr_2I_3N_6O_2$: 1215.7227; found: 1215.7243.

UV-vis (toluene): λ_{max} (nm) (log ε (dm^3 mol^{-1} cm^{-1})) = 577 (4.96), 559 (sh), 536 (sh), 324 (4.59), 270 (4.78).

FT-IR (ATR): ν (cm^{-1}) = 3497, 3061, 1738, 1601, 1548, 1494, 1436, 1263, 1240, 1177, 1141, 1087, 1042, 1023, 1001, 880, 818, 770, 744, 705, 600, 590, 518.

m-3b *(second eluted diastereomer from column chromatography on silica gel)*

^{1}**H NMR** (500 MHz, chloroform-d_1): δ (ppm) = 8.97 (d, J = 1.0 Hz, 3H), 8.34 (d, J = 8.0 Hz, 3H), 8.12-8.10 (m, 4H), 7.71 (s, 1H), 7.61 (d, J = 8.0 Hz, 1H), 7.48 (d, J = 8.0 Hz, 1H), 7.31 (ddd, J_1 = 8.0 Hz, J_2 = 6.8 Hz, J_3 = 1.0 Hz, 1H), 7.17 (ddd, J_1 = 8.0 Hz, J_2 = 6.8 Hz, J_3 = 1.0 Hz, 1H), 7.11 (ddd, J_1 = 8.5 Hz, J_2 = 6.8 Hz, J_3 = 1.0 Hz, 1H), 6.98 (ddd, J_1 = 8.5 Hz, J_2 = 6.8 Hz, J_3 = 1.0 Hz, 1H), 6.57 (d, J = 8.5 Hz, 1H), 6.48 (d, J = 8.5 Hz, 1H), 5.18 (s, 1H).

^{13}**C NMR** (125.7 MHz, chloroform-d_1) : δ (ppm) = 151.14, 150.33, 147.33, 145.31, 138.60, 133.14, 132.67, 132.42, 132.12, 132.03, 132.02, 131.44, 130.68, 129.65, 127.28, 126.97, 126.94, 126.58, 126.38, 125.99, 125.45, 124.66, 123.40, 122.74, 116.94, 116.46, 113.84, 96.12.

^{11}**B NMR** (160.4 MHz, chloroform-d_1): δ (ppm) = -14.88.

MS (MALDI-TOF, DCTB): m/z = 1215.7 $[M]^+$.

HR-MS (MALDI-TOF, DCTB + PPGNa 1000): m/z calculated for $C_{44}H_{20}BBr_2I_3N_6O_2$: 1215.7227; found: 1215.7239.

UV-vis (toluene): λ_{max} (nm) (log ε (dm^3 mol^{-1} cm^{-1})) = 577 (4.96), 559 (sh), 536 (sh), 323 (4.65), 268 (4.85).

FT-IR (ATR): ν (cm^{-1}) = 3492, 3060, 1731, 1600, 1550, 1497, 1436, 1262, 1239, 1177, 1141, 1087, 1042, 1023, 1001, 880, 818, 770, 744, 705, 600, 590, 518.

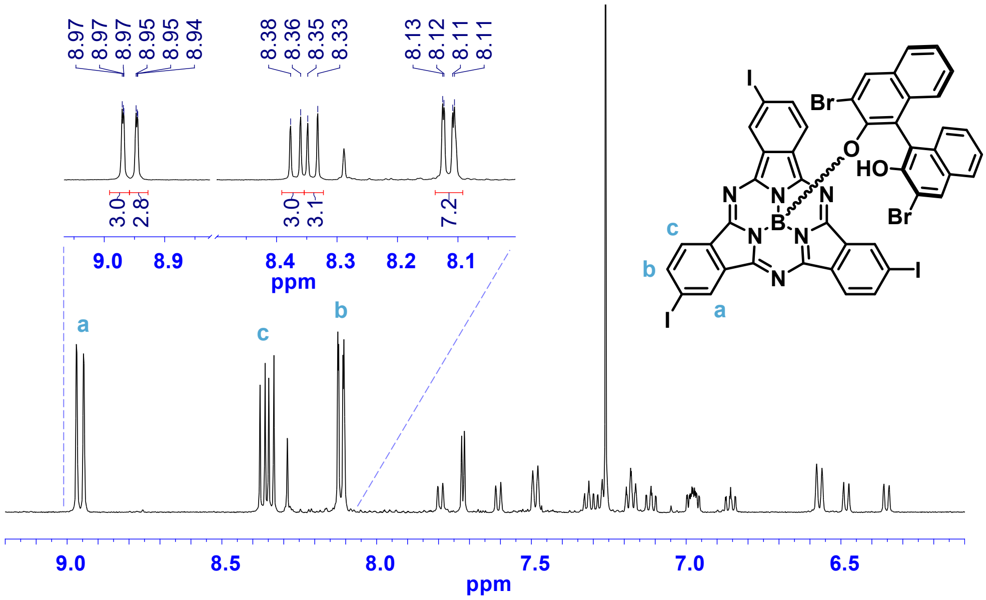

Figure S7. ^{1}H NMR spectrum (500 MHz, chloroform-d_1) of ***m*-3** purified as a mixture of diastereomers. Inset: zoom of the signals of the isoindolic protons of the SubPc macrocycle. The integral of the multiplet at 8.13- 8.11 ppm is due to the overlap of the signal of the isoindolic protons marked with “b” with the signal of an aromatic proton of the axial ligand (see Figure S8).

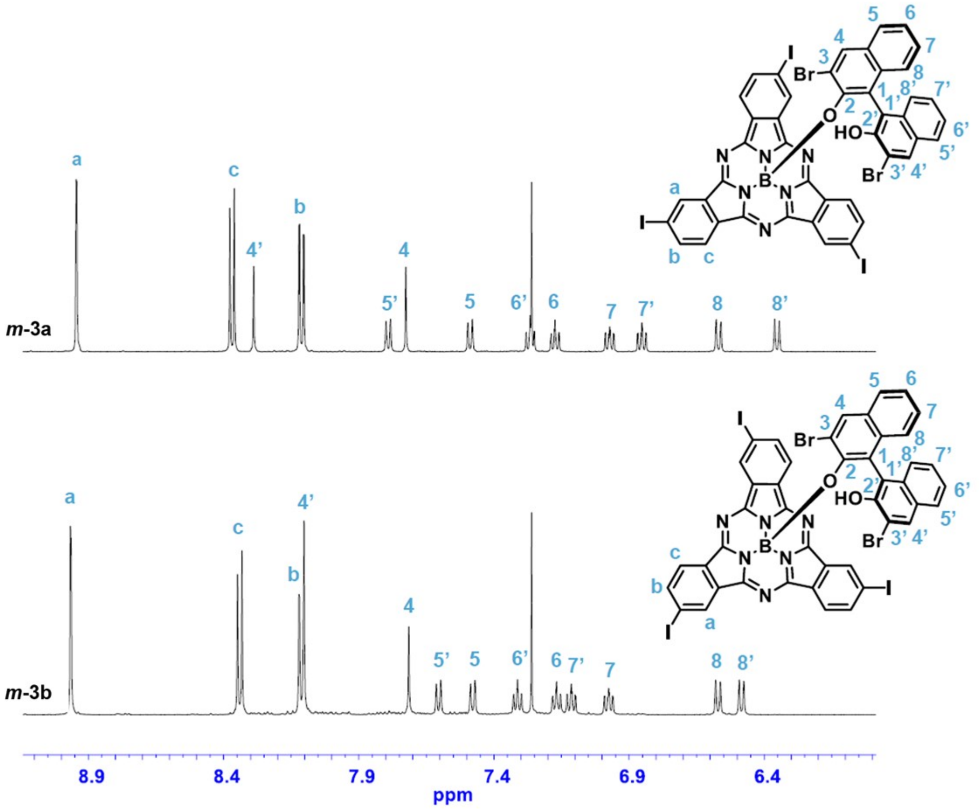

Figure S8. ^{1}H NMR spectra (500 MHz, chloroform-d_1) of ***m*-3a** and ***m*-3b**. Proton signals were assigned with the help of H-H COSY and H-H NOESY experiments.

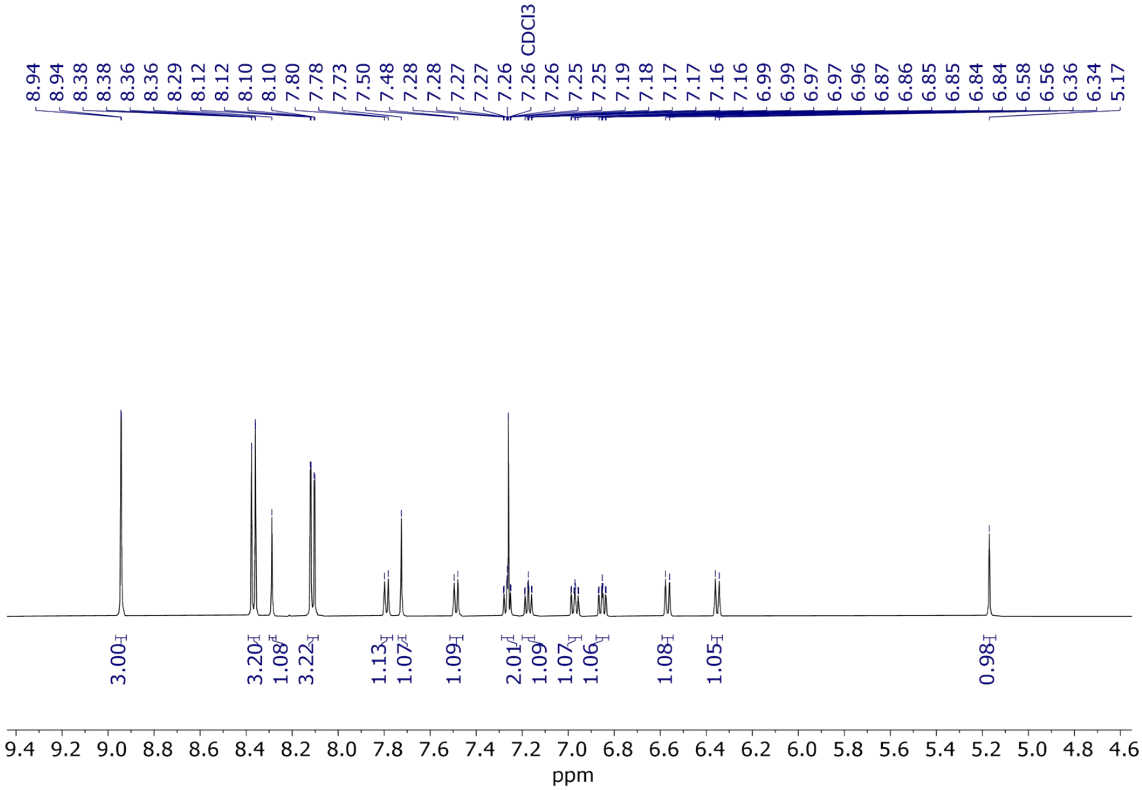

Figure S9. ^{1}H NMR spectrum (500 MHz, chloroform-d_1) of ***m*-3a**.

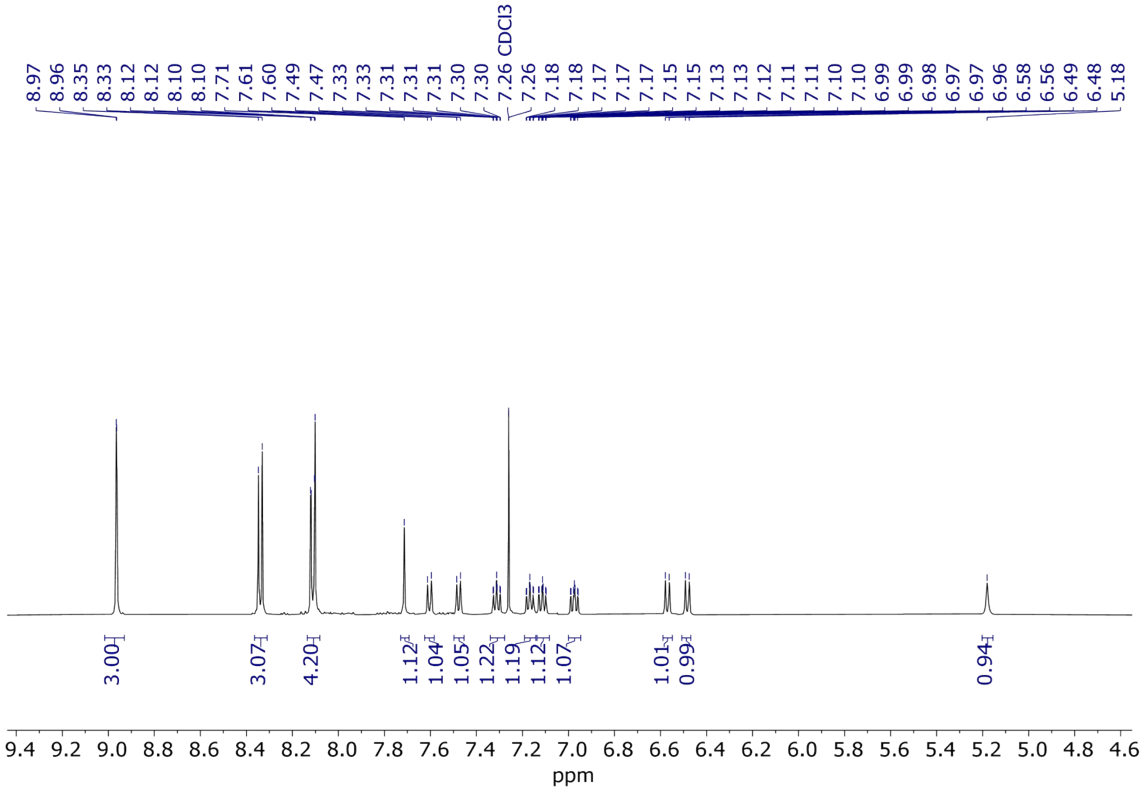

Figure S10. ^{1}H NMR spectrum (500 MHz, chloroform-d_1) of ***m*-3b**.

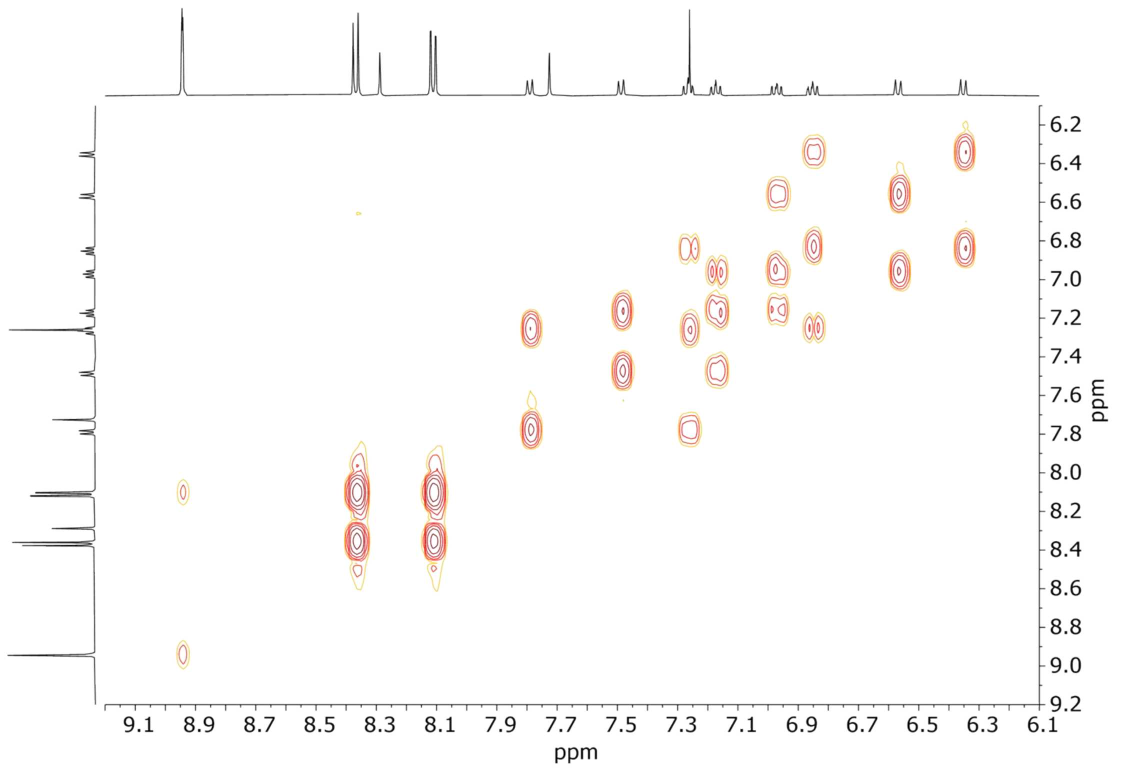

Figure S11. H-H COSY NMR spectrum (500 MHz, chloroform-d_1) of ***m*-3a**.

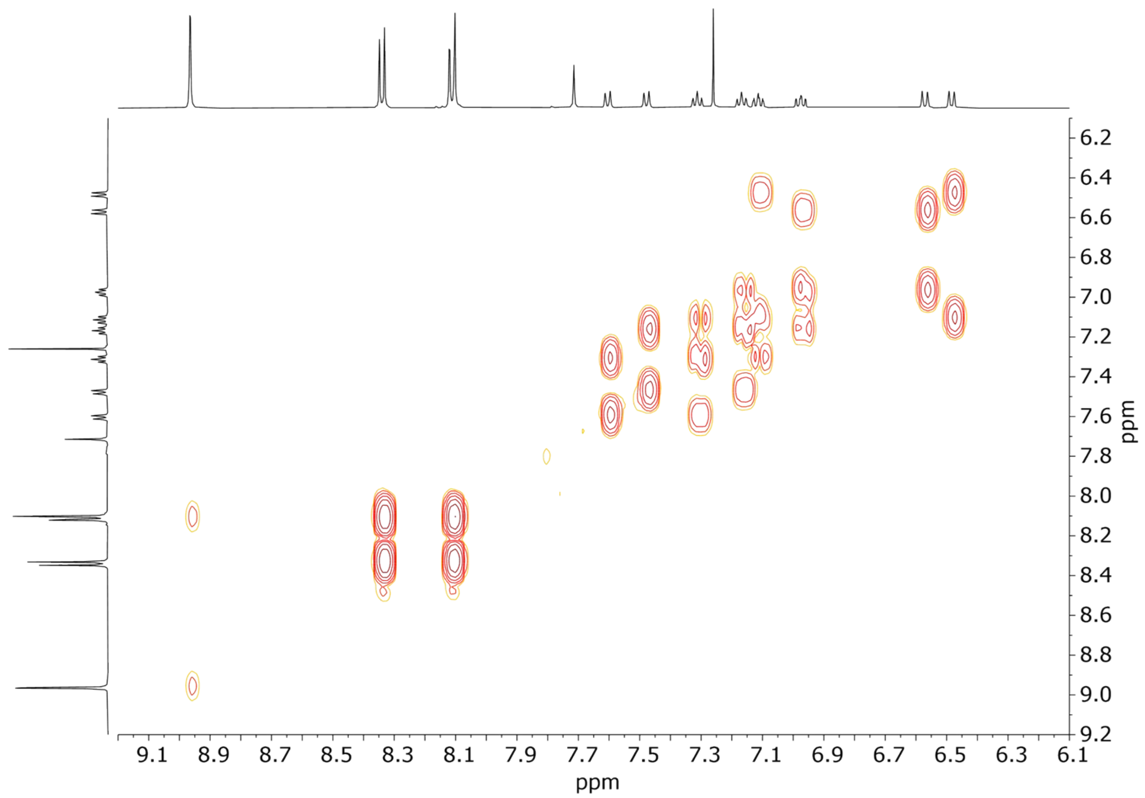

Figure S12. H-H COSY NMR spectrum (500 MHz, chloroform-d_1) of ***m*-3b**.

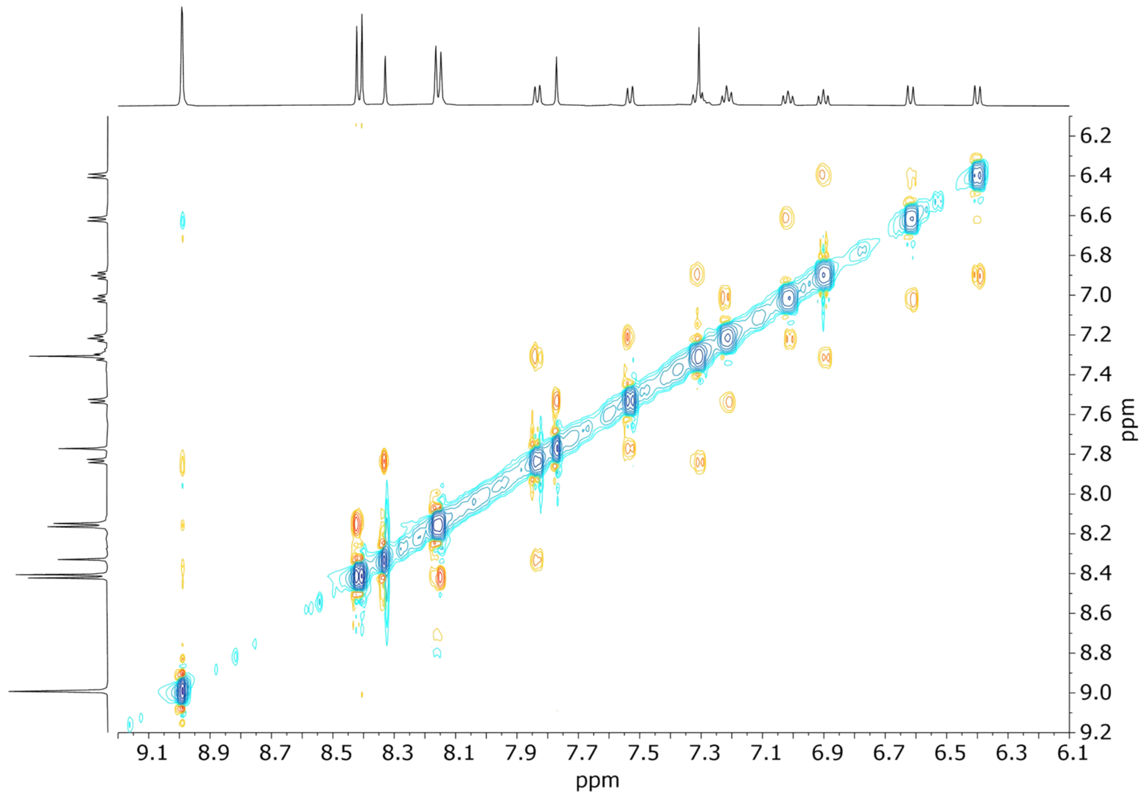

Figure S13. H-H NOESY NMR spectrum (500 MHz, chloroform-d_1) of ***m*-3a**.

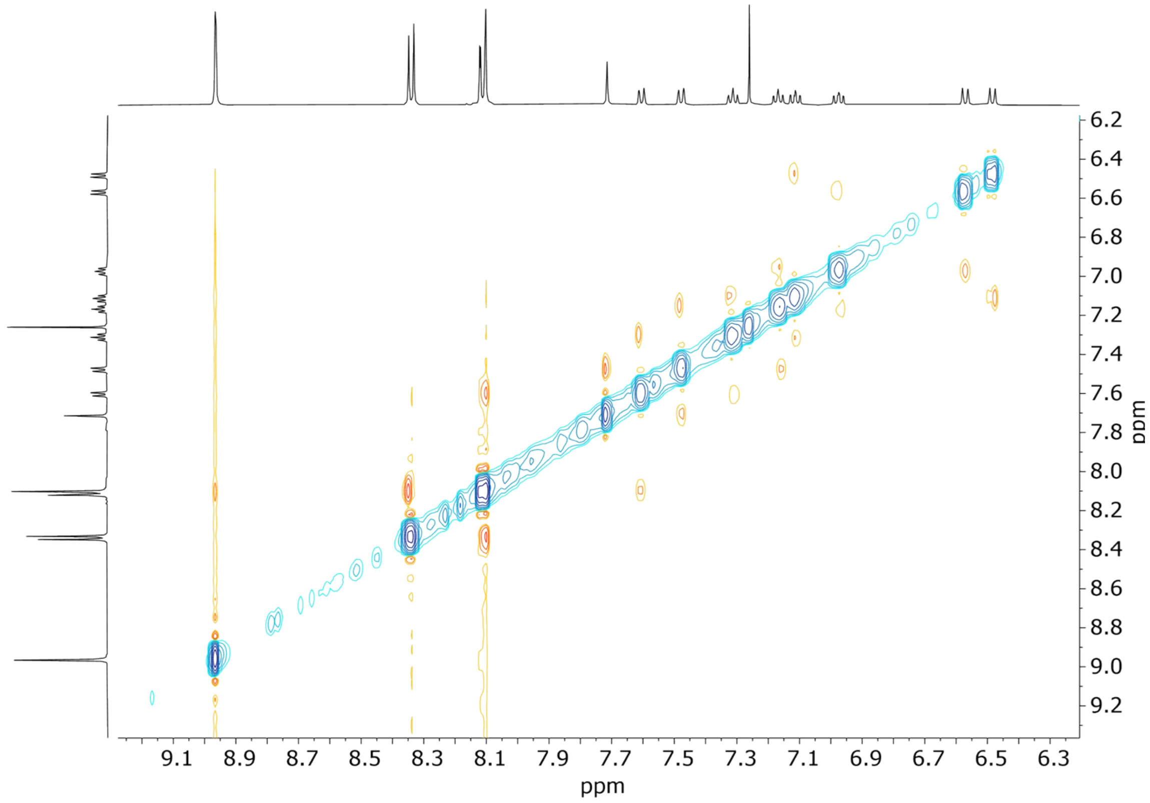

Figure S14. H-H NOESY NMR spectrum (500 MHz, chloroform-d_1) of ***m*-3b**.

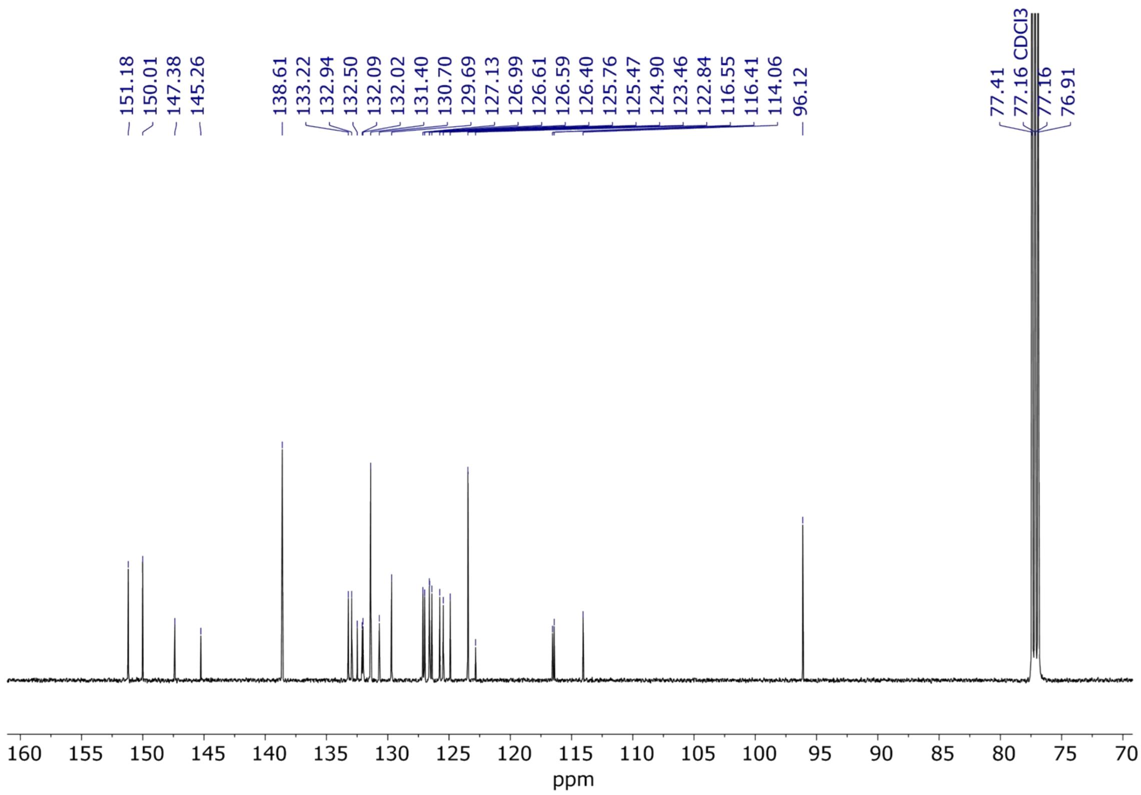

Figure S15. ^{13}C NMR spectrum (125.7 MHz, chloroform-d_1) of ***m*-3a**.

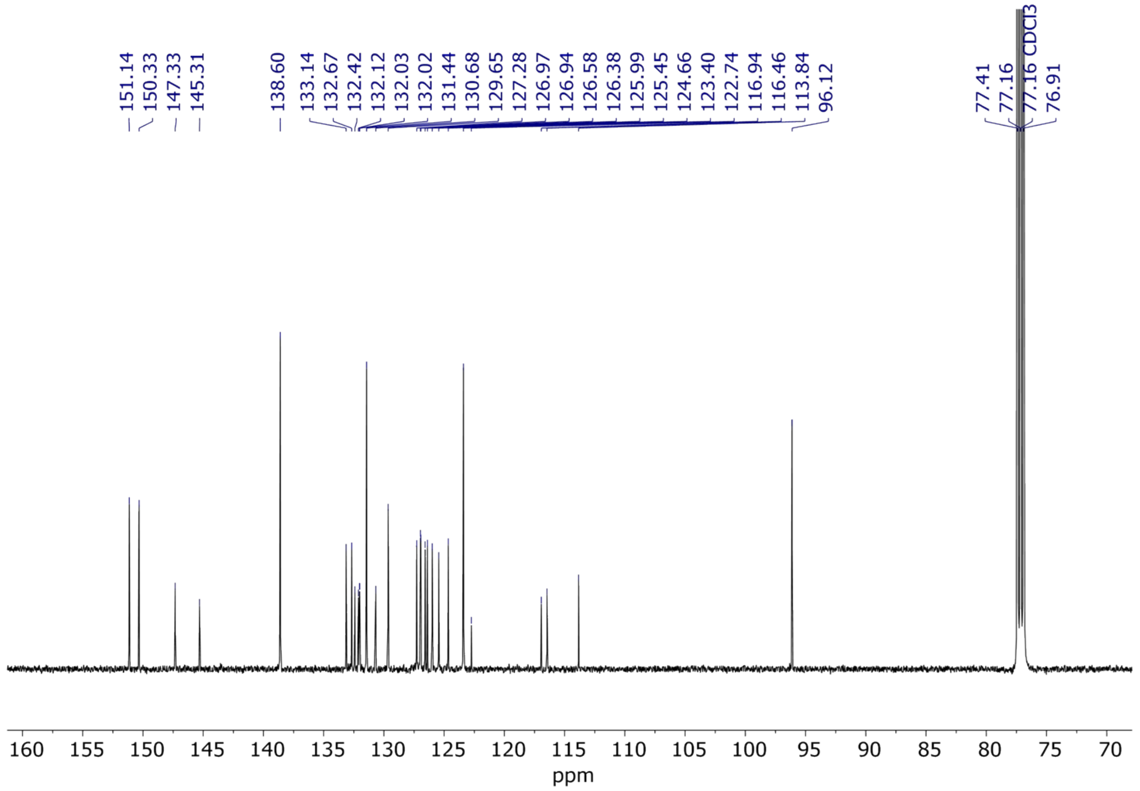

Figure S16. ^{13}C NMR spectrum (125.7 MHz, chloroform-d_1) of ***m*-3b**.

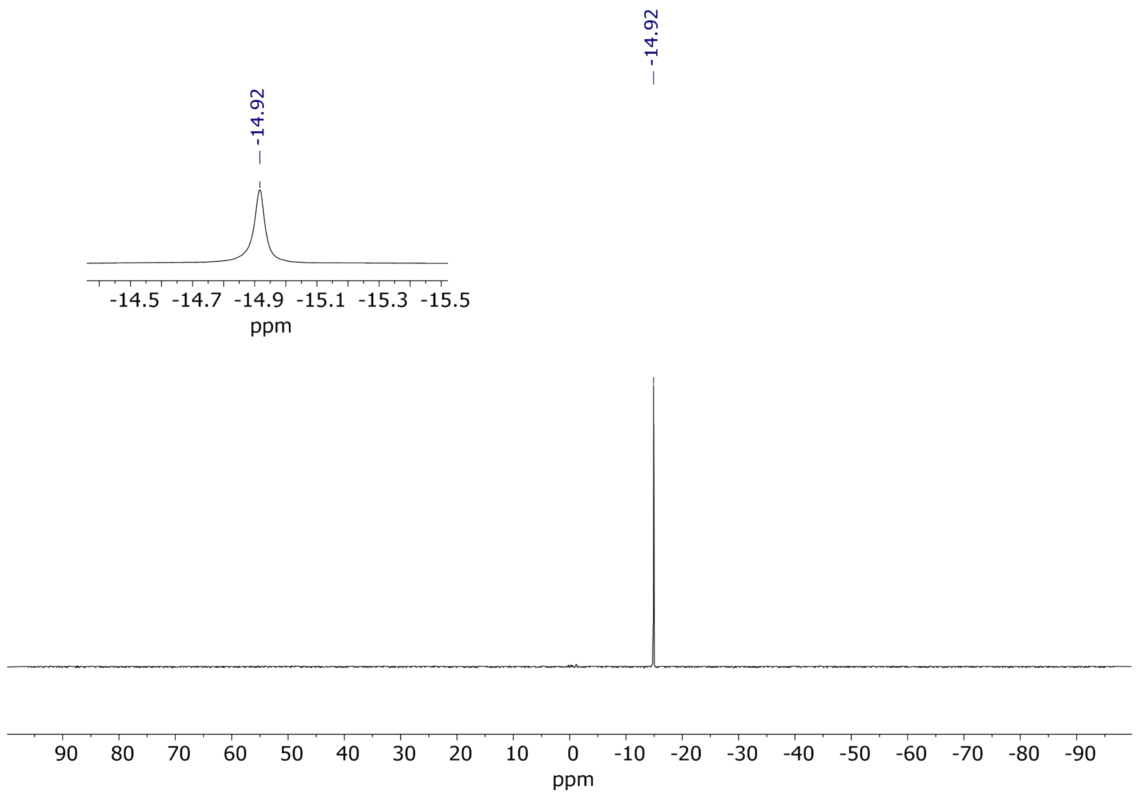

Figure S17. ^{11}B NMR spectrum (160.4 MHz, chloroform-d_1) of ***m*-3a**.

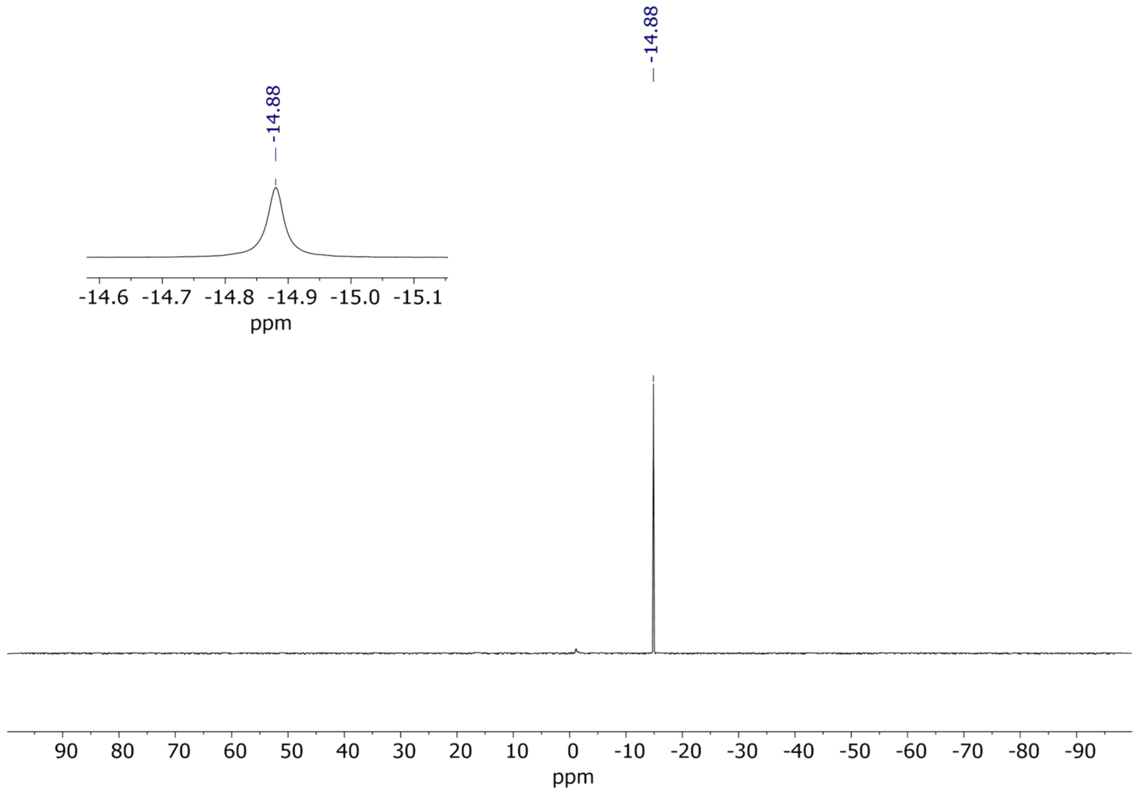

Figure S18. ^{11}B NMR spectrum (160.4 MHz, chloroform-d_1) of ***m*-3b**.

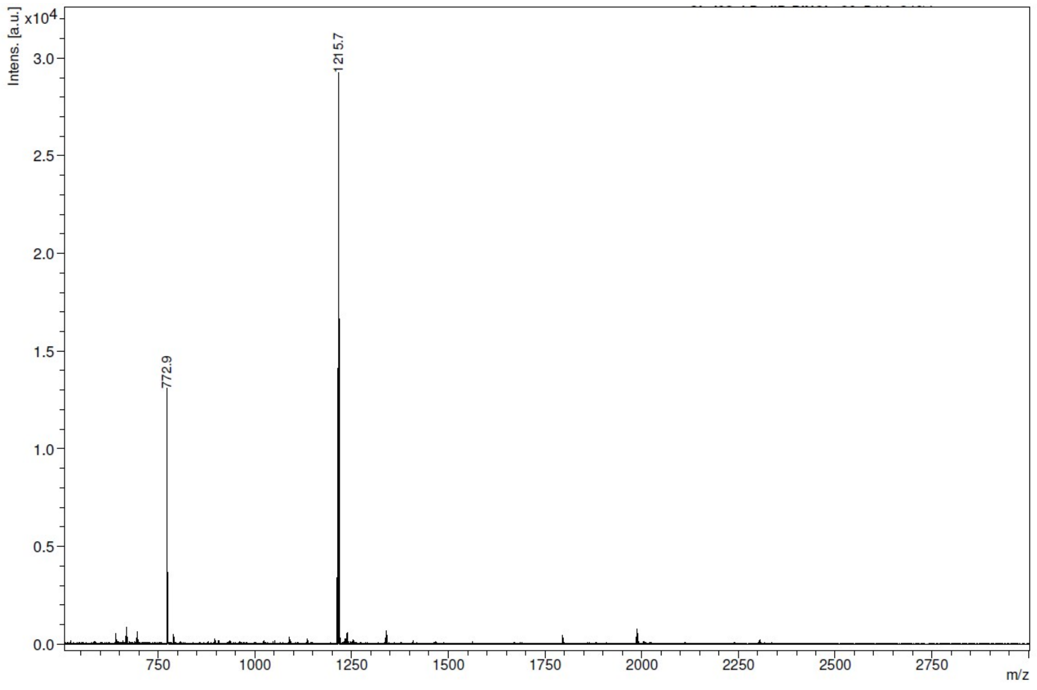

Figure S19. MALDI-TOF mass spectrum (matrix: DCTB) of ***m*-3a**.

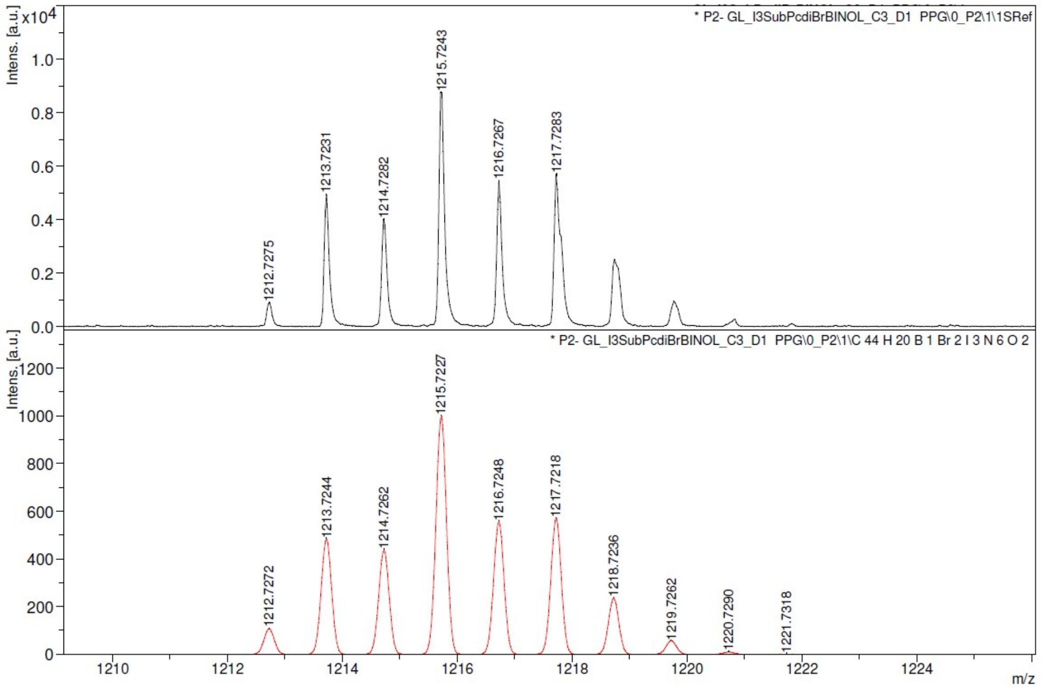

Figure S20. (Top) HR-MS (MALDI-TOF) spectrum (matrix: DCTB + PPGNa 1000) and (bottom) calculated isotopic pattern of ***m*-3a**.

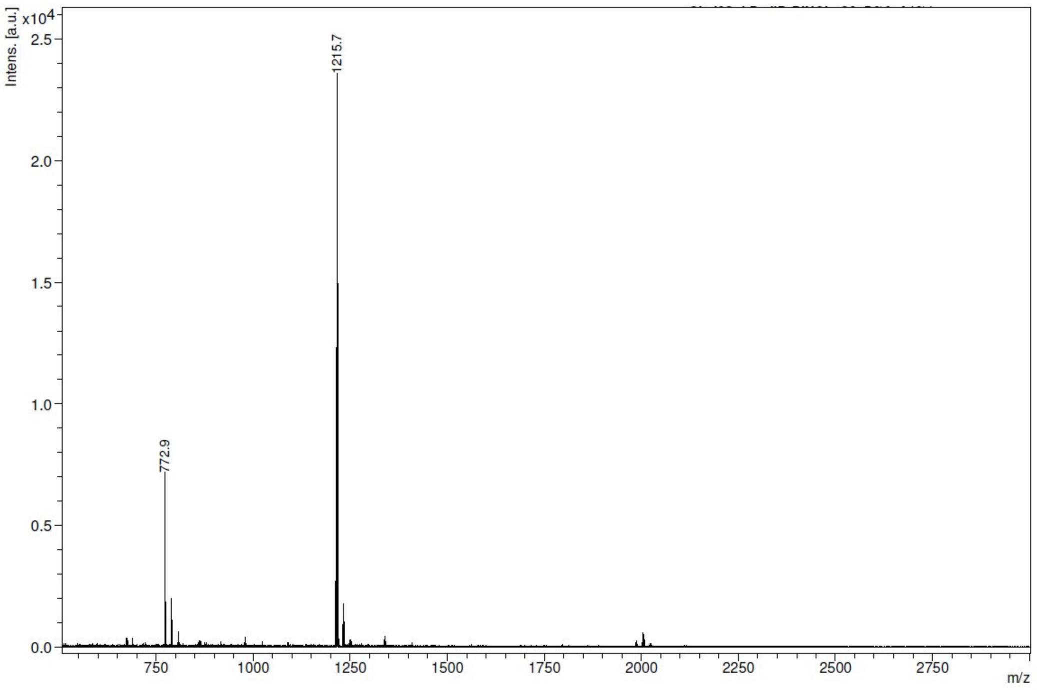

Figure S21. MALDI-TOF mass spectrum (matrix: DCTB) of ***m*-3b**.

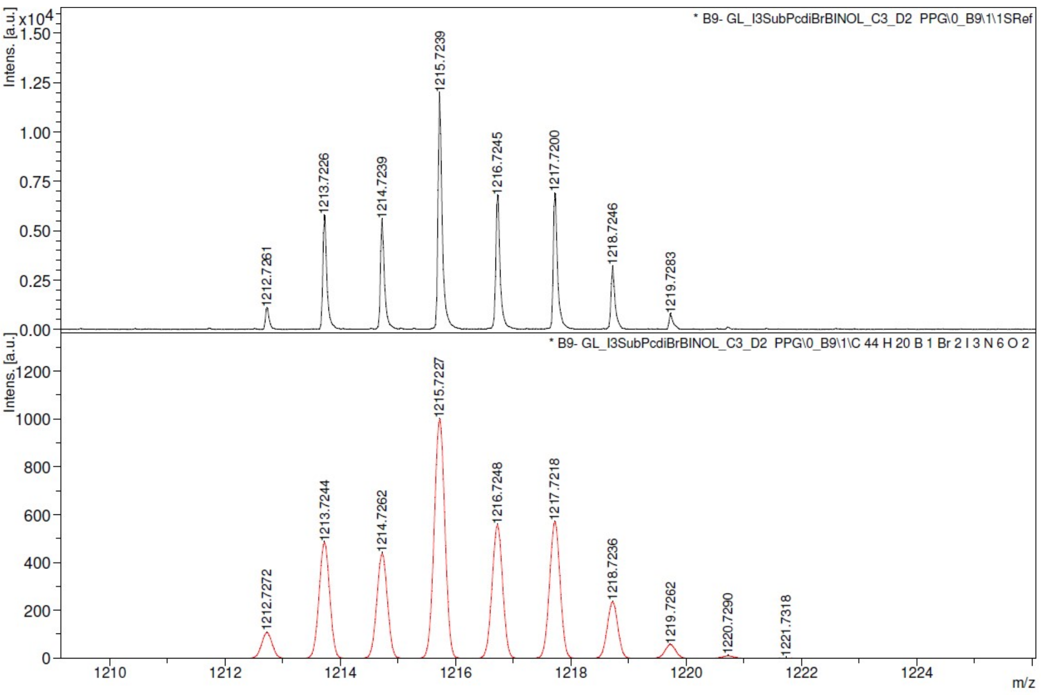

Figure S22. (Top) HR-MS (MALDI-TOF) spectrum (matrix: DCTB + PPGNa 1000) and (bottom) calculated isotopic pattern of ***m*-3b**.

Synthesis and characterization of *o*-2

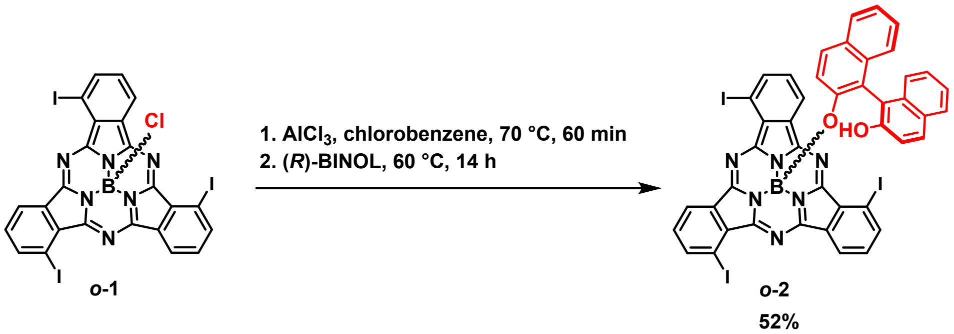

Scheme S3. Synthesis of ***o*-2** as a mixture of diastereomers from racemic ***o*-1** *via* activation of the axial position with $AlCl_3$.

I_3SubPc-Cl ***o*-1** (135.0 mg, 0.16 mmol) and $AlCl_3$ (32 mg, 0.24 mmol) were dissolved in anhydrous chlorobenzene (2 mL) under argon atmosphere in a Schlenk flask and the reaction mixture was stirred at 70 °C for 60 min. Then, (*R*)-(+)-1,1′-bi-2-naphthol (460 mg, 1.6 mmol) was added, and the mixture was stirred at 60 °C for 14 h. The reaction mixture was then allowed to cool down to room temperature, poured onto brine (60 mL) and extracted with ethyl acetate (3x30 mL) The organic layer was collected, washed with water (2x30 mL) and dried over anhydrous $MgSO_4$. After filtration of the drying agent, the solvent was evaporated under reduced pressure and the residue was precipitated from a methanol/water 8:1 (*v*/*v*) mixture. The precipitate was collected by filtration and subjected to column chromatography on silica gel (6 cm ø × 22 cm) using toluene as eluent. ***o*-2a** (first eluted diastereomer) and ***o*-2b** (second eluted diastereomer) were collected separately. Recrystallization from a dichloromethane/MeOH/water mixture afforded the isolated ***o*-2a** and ***o*-2b** diastereomers as purple solids in 24% yield. The overall yield of ***o*-2** is 52% (92 mg).

***o*-2a** *(first eluted diastereomer from column chromatography on silica gel)*

^{1}H NMR (500 MHz, chloroform-d_1): δ (ppm) = 8.69 (d, J = 7.5 Hz, 3H), 8.27 (d, J = 7.5 Hz, 3H), 7.91 (d, J = 8.5 Hz, 1H), 7.67 (d, J = 7.5 Hz, 1H), 7.61 (d, J = 8.5 Hz, 1H), 7.50 (t, J = 7.5 Hz, 3H), 7.41 (d, J = 8.5 Hz, 1H), 7.16 (t, J = 7.5 Hz, 1H), 7.08 (d, J = 8.5 Hz, 1H), 7.05-7.00 (m, 3H), 6.77 (d, J = 8.5 Hz, 1H), 6.37 (d, J = 8.5 Hz, 1H), 5.65 (d, J = 8.5 Hz, 1H), 4.46 (s, 1H).

^{13}C NMR (125.7 MHz, chloroform-d_1) : δ (ppm) = 151.0, 150.9, 149.1, 148.2, 140.9, 138.0, 133.4, 132.9, 132.5, 132.2, 130.3, 130.3, 130.2, 129.9, 129.2, 128.8, 128.3, 128.0, 127.9, 126.4, 126.2, 126.1, 125.4, 124.8, 124.5, 123.2, 122.2, 120.8, 120.5, 118.4, 114.7.

MS (MALDI-TOF, DCTB): m/z = 1057.9 [M]$^+$.

HR-MS (MALDI-TOF, DCTB + PPGNa 1000): m/z calculated for $C_{44}H_{22}BI_3N_6O_2$: 1057.9033; found: 1057.9039.

UV-vis (chloroform): λ_{max} (nm) (log ε (dm^3 mol^{-1} cm^{-1})) = 577 (4.85), 533 (sh), 281 (4.55).

FT-IR (ATR): ν (cm^{-1}) = 3500, 3426, 3055, 2918, 2848, 1730, 1618, 1595, 1546, 1502, 1451, 1422, 1389, 1331, 1253, 1204, 1167, 1135, 1082, 1025, 902, 814, 791, 749, 703, 589.

***o*-2b** *(second eluted diastereomer from column chromatography on silica gel)*

^{1}H NMR (500 MHz, chloroform-d_1): δ (ppm) = 8.74 (d, J = 7.5 Hz, 3H), 8.26 (d, J = 7.5 Hz, 3H), 7.96 (d, J = 8.5 Hz, 1H), 7.81 (d, J = 8.5 Hz, 1H), 7.64 (d, J = 7.5 Hz, 1H), 7.50 (t, J = 7.5 Hz, 3H), 7.44 (d, J = 8.5 Hz, 1H), 7.20 (t, J = 7.5 Hz, 1H), 7.10 (d, J = 8.5 Hz, 1H), 7.00 (t, J = 7.5 Hz, 1H), 6.95 (t, J = 7.5 Hz, 1H), 6.74 (d, J = 7.5 Hz, 2H), 6.36 (d, J = 8.5 Hz, 1H), 5.64 (d, J = 8.5 Hz, 1H), 4.51 (s, 1H).

^{13}C NMR (125.7 MHz, chloroform-d_1) : δ (ppm) = 151.1, 151.0, 149.05, 148.0, 140.9, 133.6, 132.8, 132.5, 132.1, 130.3, 130.2, 130.1, 128.6, 128.3, 127.9, 126.3, 126.1, 124.7, 124.5, 123.1, 122.2, 120.7, 118.3, 114.6.

MS (MALDI-TOF, DCTB): m/z = 1057.9 [M]$^+$.

UV-vis (chloroform): λ_{max} (nm) (log ε (dm^3 mol^{-1} cm^{-1})) = 577 (4.88), 533 (sh), 281 (4.68).

FT-IR (ATR): ν (cm^{-1}) = 3512, 3428, 3052, 2924, 2852, 1729, 1618, 1595, 1546, 1507, 1451, 1422, 1388, 1331, 1253, 1204, 1167, 1134, 1084, 1025, 904, 814, 790, 772, 749, 705, 641, 590.

Note: The reaction conditions whereby the (*R*)-BINOL ligand is incorporated at the axial position of the SubPc macrocycle are a crucial aspect. When the axial ligand exchange reaction is carried out by heating the mixture of the SubPc-Cl and the phenol in a high boiling point solvent (namely chlorobenzene) in the presence of a base (i.e. DBU), racemization of the chiral derivatizing agent is observed (Scheme S4). In this case, single crystal X-ray diffraction analysis of the first eluted fraction from column chromatography on silica gel evidenced the presence of both *R*-BINOL and *S*-BINOL at the apical position of the SubPc scaffold (Section 5.1). Accordingly, the lack of CD signal of the two fractions collected by column chromatography further confirm that racemization of the BINOL derivative takes place in the reaction conditions employed. In particular, each one of the two fractions collected after column chromatography contains a mixture of enantiomer. In particular, one fraction contains *R*-BINOL/*M*-SubPc + *S*-BINOL/*P*-SubPc (as evidenced by X-ray diffraction analysis), and the other fraction will likely contain *R*-BINOL/*P*-SubPc + *S*-BINOL/*M*-SubPc.

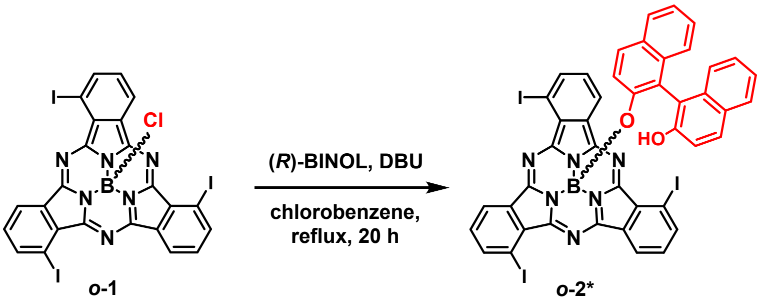

Scheme S4. Rection scheme and conditions attempted for the synthesis of ***o*-2*** without activation of the SubPc apical position, leading to the racemization of the BINOL chiral auxiliary.

Further evidence for racemization of the BINOL derivative in refluxing chlorobenzene in the presence of a base come from specific rotation measurements. In this connection, a test was performed by refluxing (*R*)-BINOL in chlorobenzene for 20 hours. The specific rotation of the resulting product remained unchanged (*i.e.*, $[\alpha]_D^{20} = +36°$, c = 1.0 g/100 ml in THF). Nevertheless, upon refluxing (*R*)-BINOL and 1,8-diazabiciclo[5.4.0]undec-7-ene (DBU) in chlorobenzene for 20 hours, the specific rotation dropped to zero, evidencing a full racemization of the molecule ($[\alpha]_D^{20} = +36°$, c = 1.0 g/100 ml in THF). Therefore, the presence of a base appears to be crucial for such undesirable process.

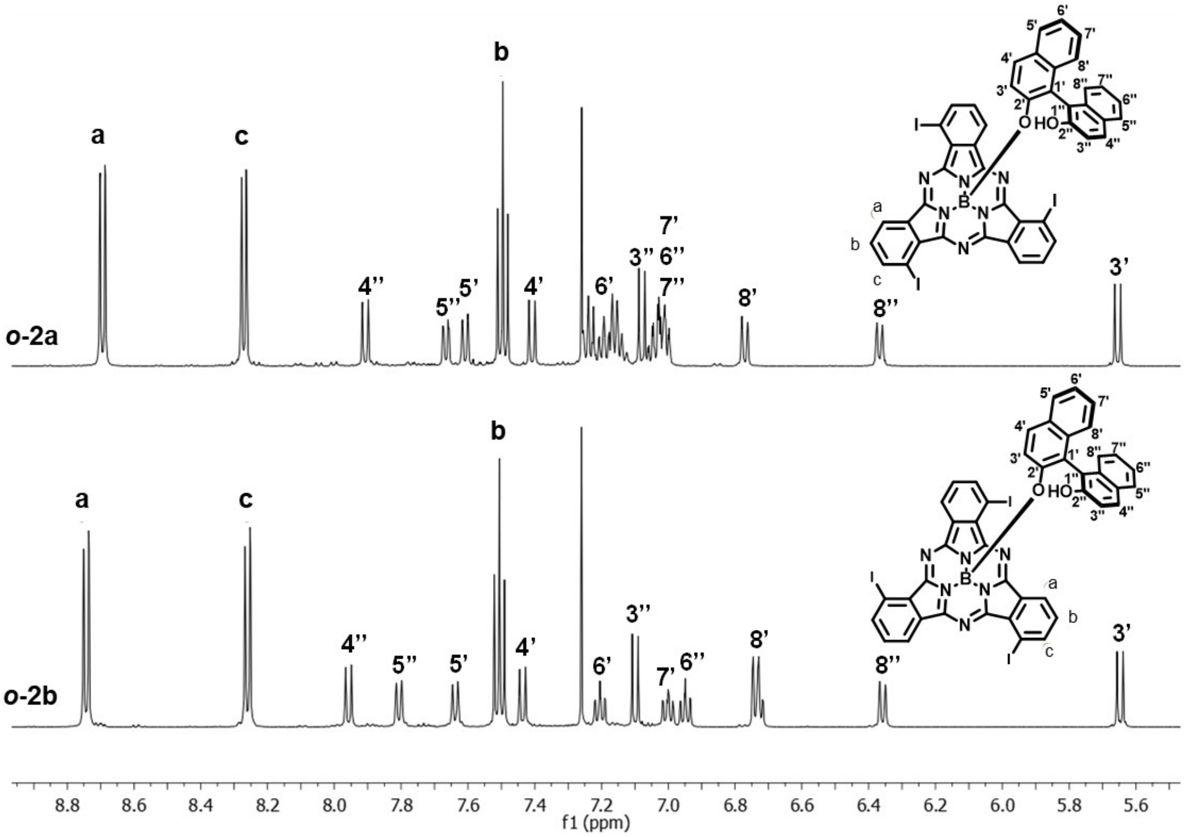

Figure S23. ^{1}H NMR spectra (500 MHz, chloroform-d_1) of ***o*-2a** and ***o*-2b**. Proton signals were assigned with the help of H-H COSY and H-H NOESY experiments. The absolute configuration of the SubPc macrocycle is not known. For visualization purposes, the *M* configuration has been depicted for ***o*-2a** and the *P* configuration has been depicted for ***o*-2b** .

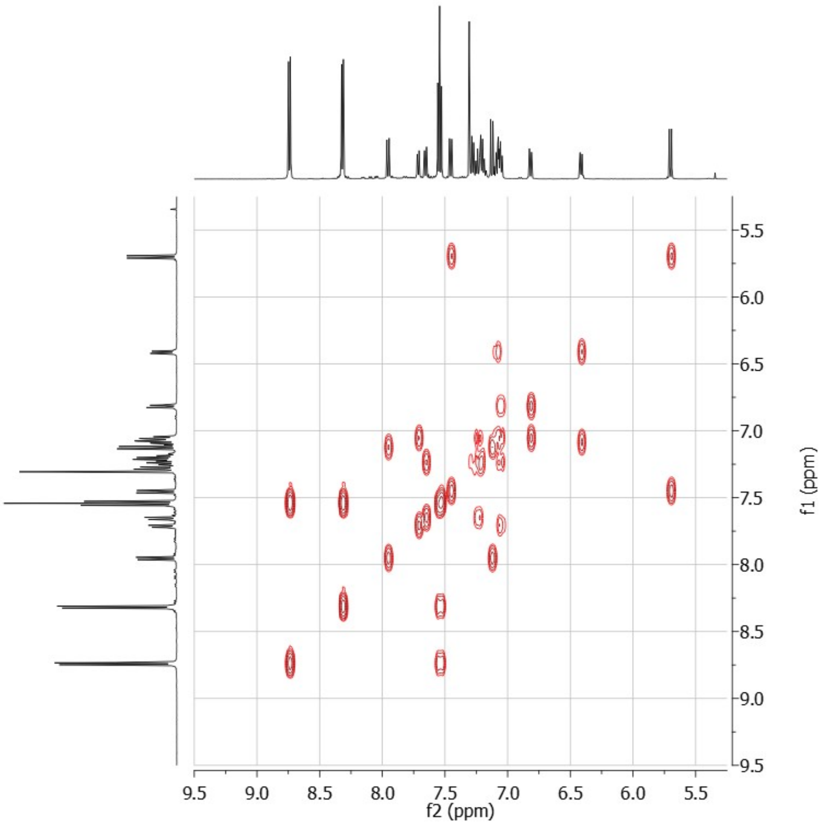

Figure S24. H-H COSY NMR spectrum (500 MHz, chloroform-d_1) of ***o*-2a**.

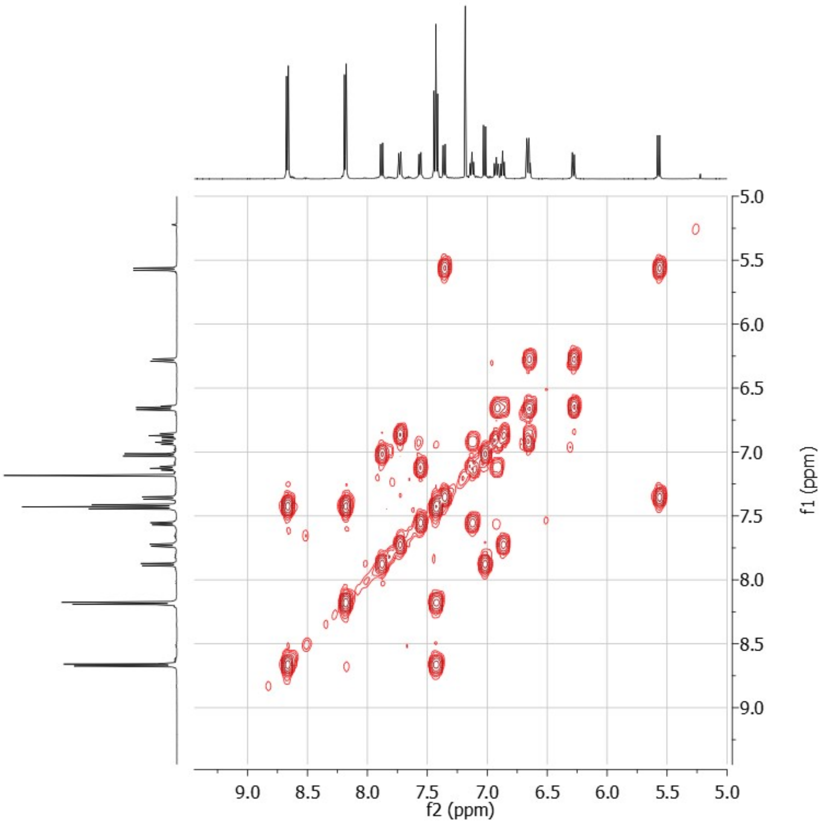

Figure S25. H-H COSY NMR spectrum (500 MHz, chloroform-d_1) of ***o*-2b**.

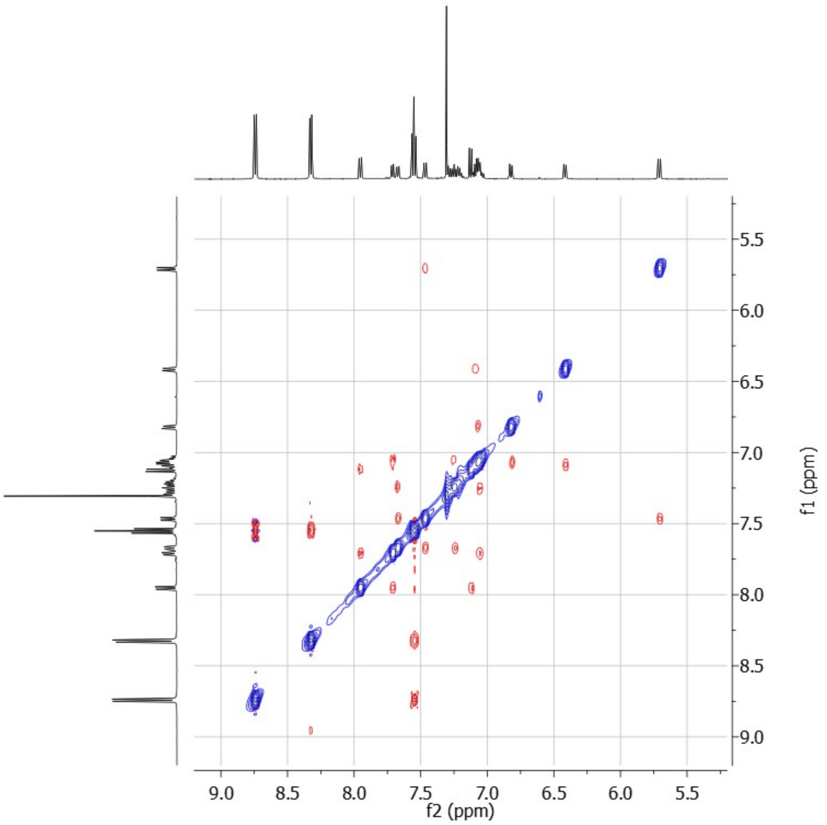

Figure S26. H-H NOESY NMR spectrum (500 MHz, chloroform-d_1) of ***o*-2a**.

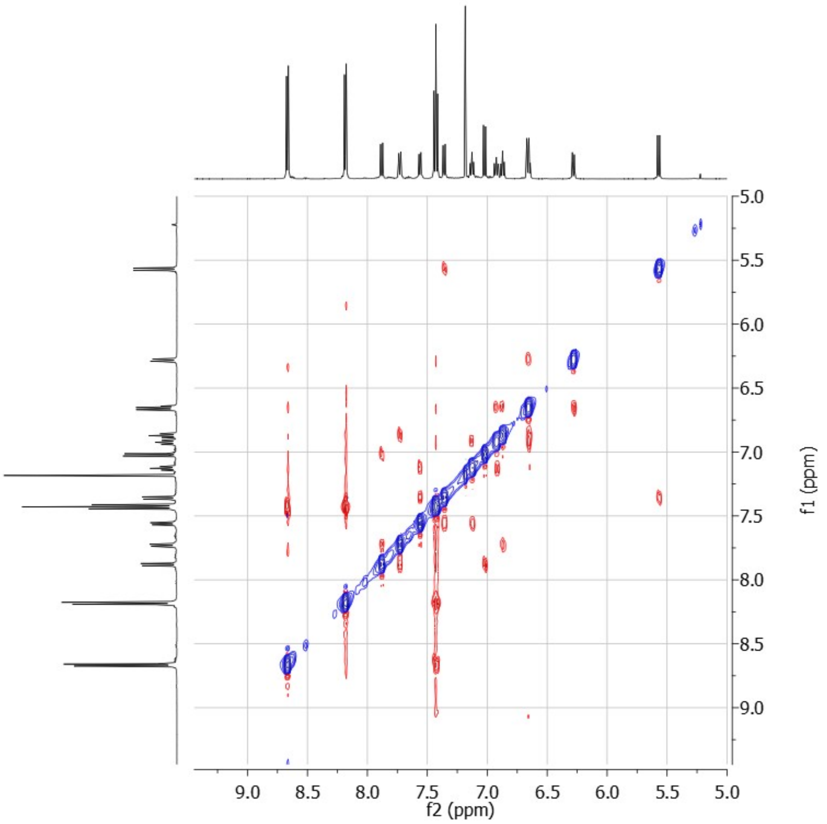

Figure S27. H-H NOESY NMR spectrum (500 MHz, chloroform-d_1) of ***o*-2b**.

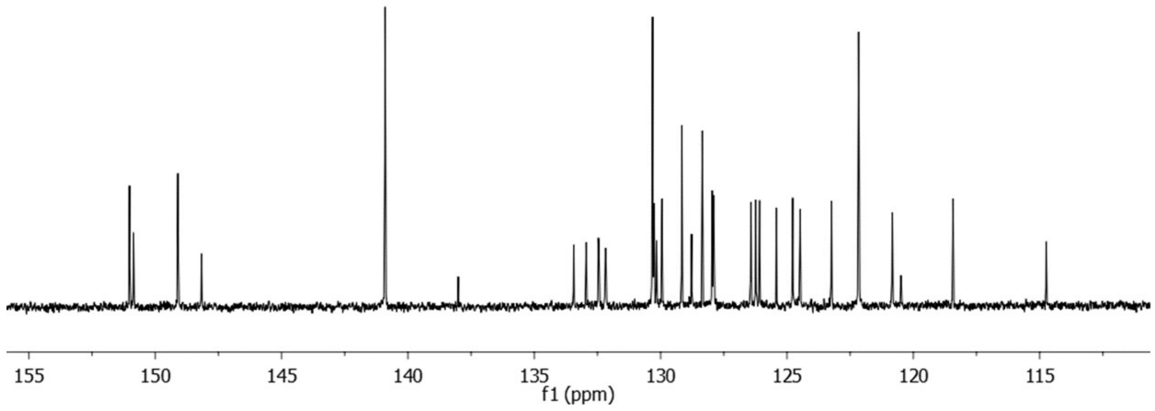

Figure S28. ^{13}C NMR spectrum (125.7 MHz, chloroform-d_1) of ***o*-2a**.

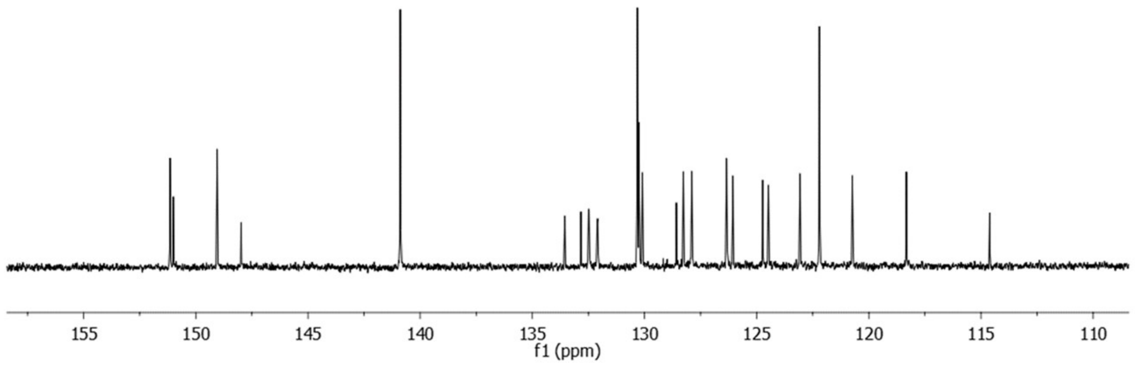

Figure S29. ^{13}C NMR spectrum (125.7 MHz, chloroform-d_1) of ***o*-2b**.

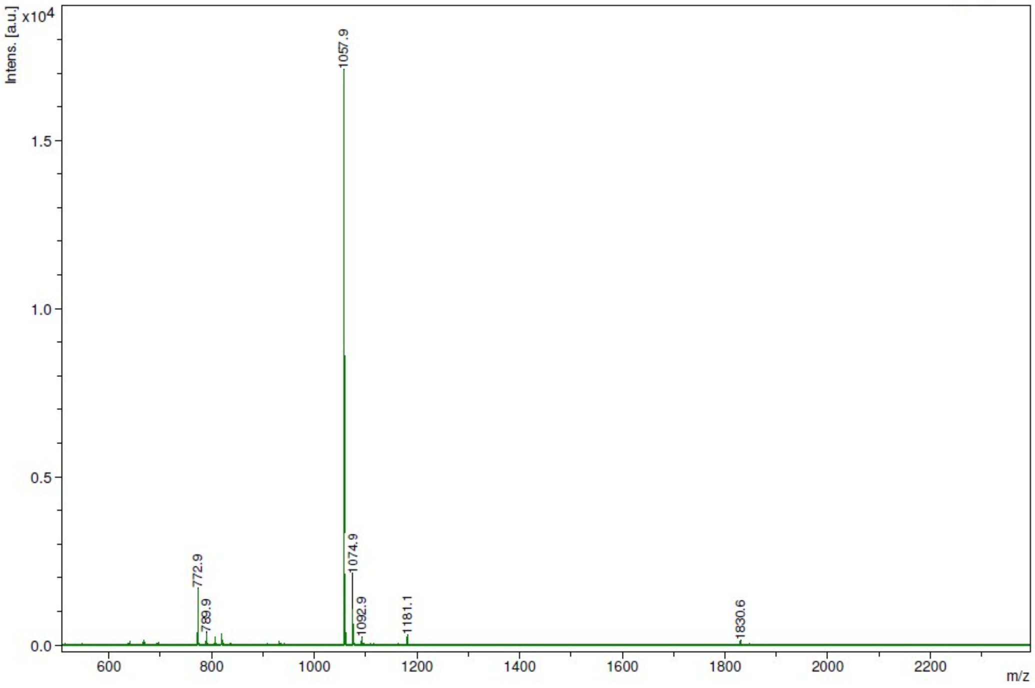

Figure S30. MALDI-TOF mass spectrum (matrix: DCTB) of ***o*-2a**.

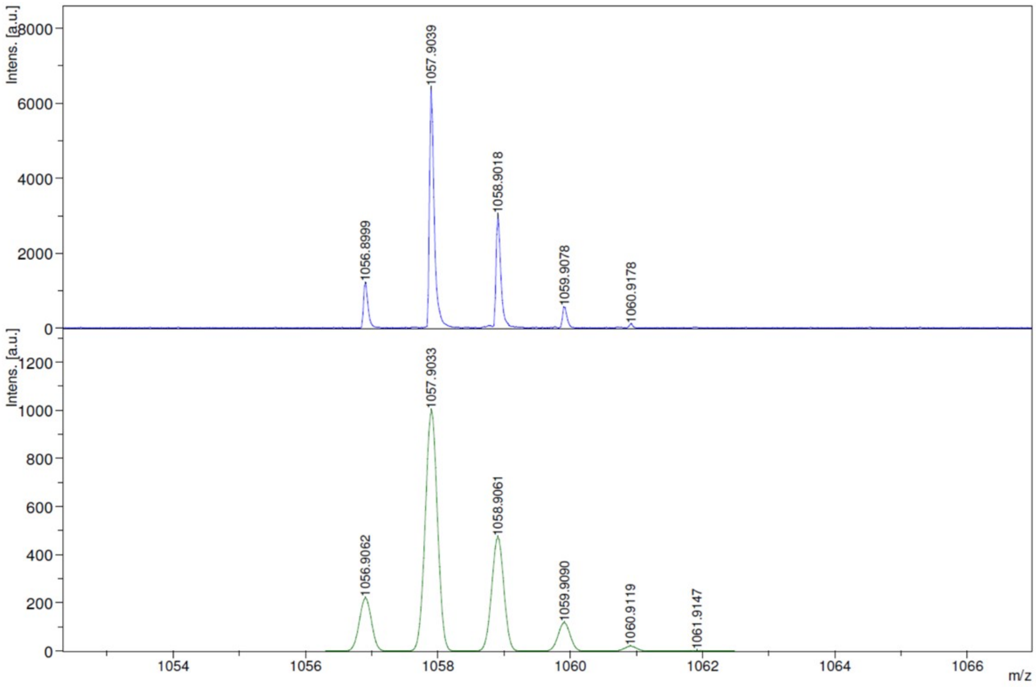

Figure S31. (Top) HR-MS (MALDI-TOF) spectrum (matrix: DCTB + PPGNa 1000) and (bottom) calculated isotopic pattern of ***o*-2a**.

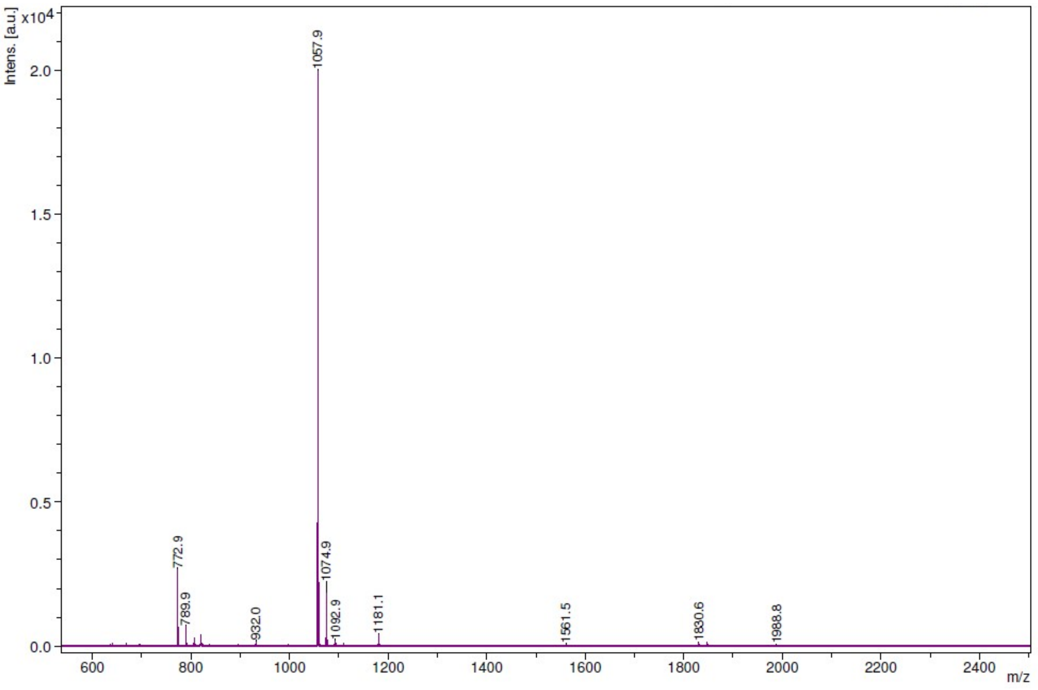

Figure S32. MALDI-TOF mass spectrum (matrix: DCTB) of ***o*-2b**.

3. Synthetic procedures for the removal of the chiral auxiliary from *m*-3 and *o*-2

Removal of the chiral auxiliary from *m*-3

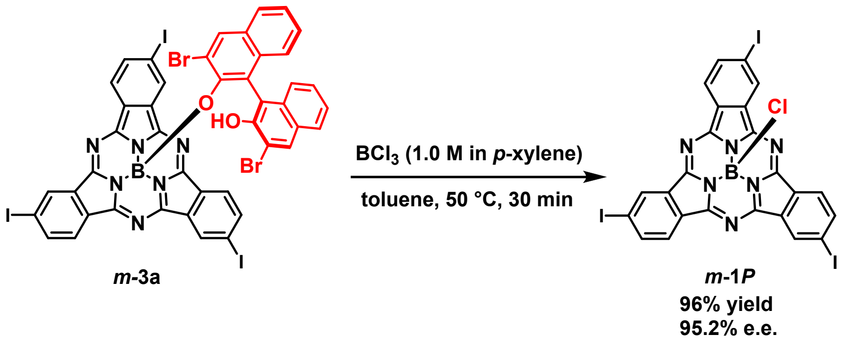

Scheme S5. Replacement of the chiral auxiliary in ***m*-3a** with a chlorine atom to afford ***m*-1*P***.

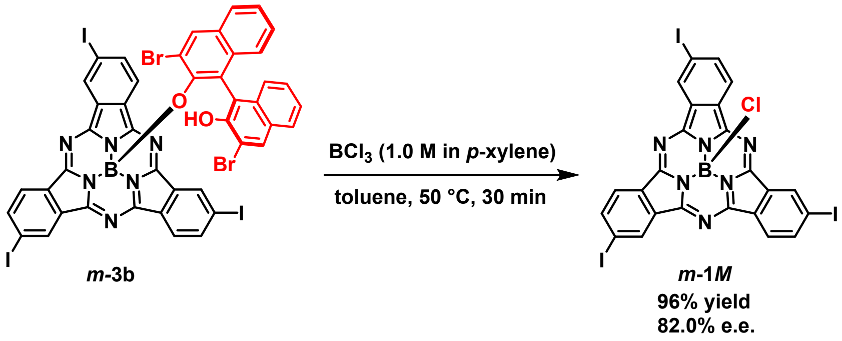

Scheme S6. Replacement of the chiral auxiliary in ***m*-3b** with a chlorine atom to afford ***m*-1*M***.

m*-3a** or ***m*-3b** (25.0 mg, 20.6 µmol) was dissolved in anhydrous toluene (2 mL) in a 10 mL round bottom flask and a 1.0 M solution of BCl_3 in *p*-xylene (206 µL, 206 µmol) was added under argon atmosphere. The reaction mixture was stirred at 50 °C for 30 min, and then allowed to cool down to room temperature and flushed with argon. After evaporating the solvent under reduced pressure, the crude was purified by a short silica plug in toluene. Recrystallization from dichloromethane/methanol afforded either ***m*-1*P (95.2% e.e.) or ***m*-1*M*** (82.0% e.e.) as a purple solid in 96% yield (16 mg).

Removal of the chiral auxiliary from *o*-2

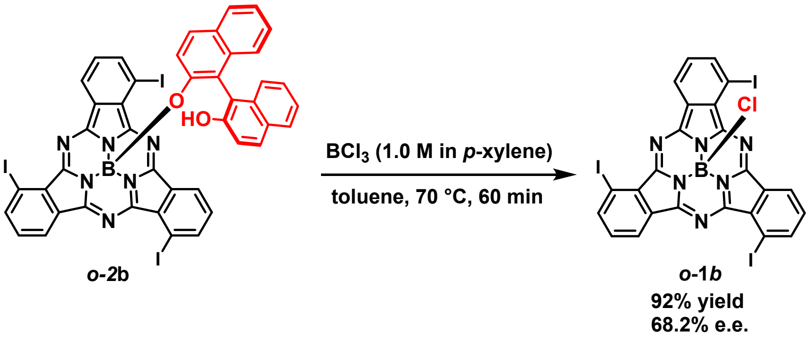

Scheme S7. Replacement of the chiral auxiliary in ***o*-2b** with a chlorine atom to afford ***o*-1b**. The absolute configuration of the SubPc macrocycle in the starting material is not known. For visualization purposes, the *P* configuration has been depicted.

***o*-2b** (10.0 mg, 9.4 μmol) was dissolved in anhydrous toluene (0.5 mL) in a 2 mL Schlenk flask and a 1.0 M solution of BCl_3 in *p*-xylene (190 μL, 190 μmol) was added under argon atmosphere. The reaction mixture was stirred at 70 °C for 1 h, and then allowed to cool down to room temperature and flushed with argon. After evaporating the solvent under reduced pressure, the crude was purified by a short silica plug in toluene. Recrystallization from dichloromethane/methanol afforded either ***o*-1b** (68.2% e.e.) as a purple solid in 92% yield (7 mg).

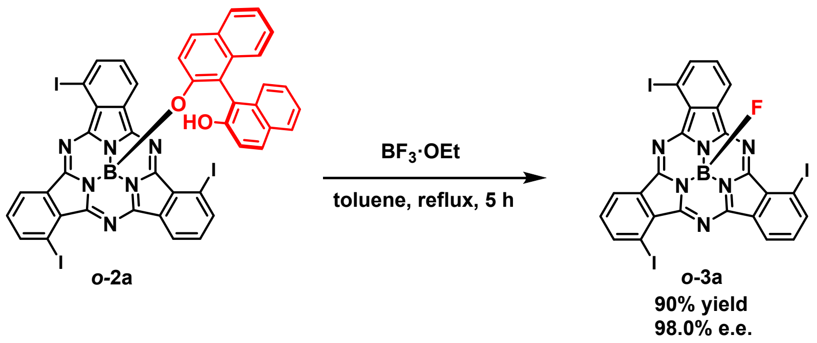

Scheme S8. Replacement of the chiral auxiliary in ***o*-2a** with a fluorine atom to afford ***o*-3a**. The absolute configuration of the SubPc macrocycle in the starting material and in the final product is not known. For visualization purposes, the *M* configuration has been depicted.

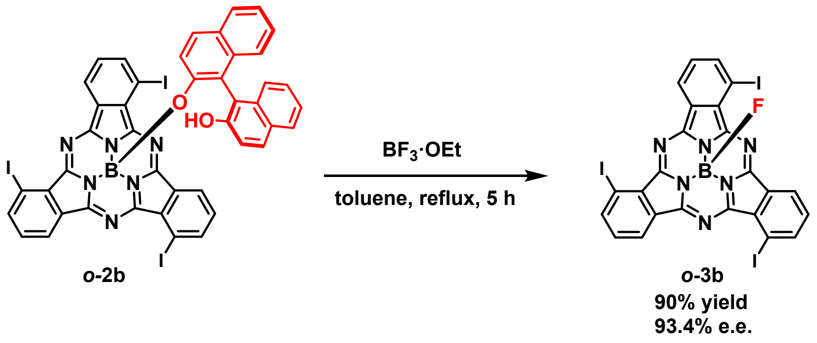

Scheme S9. Replacement of the chiral auxiliary in ***o*-2b** with a fluorine atom to afford ***o*-3b**. The absolute configuration of the SubPc macrocycle in the starting material and in the final product is not known. For visualization purposes, the *P* configuration has been depicted.

***o*-2a** or ***o*-2b** (25.0 mg, 24 μmol) was dissolved in anhydrous toluene (2.4 mL) under argon atmosphere in a 10 mL round bottom flask and $BF_3·OEt_2$ (74 μL, 0.06 mmol) was added dropwise. The reaction mixture was refluxed for 5 min, and then allowed to cool down to room temperature. After evaporating the solvent under reduced pressure, the crude was purified by column chromatography on silica gel (eluent: toluene). Recrystallization from dichloromethane/methanol afforded either ***o*-3a** (98.0% e.e.) or ***o*-3b** (93.4% e.e.) as a purple solid in 90% yield (16 mg).

^{1}H-NMR (300 MHz, Chloroform-d_1): δ (ppm) = 8.95 (d, *J* = 7.2 Hz, 3H), 8.40 (d, *J* = 7.5 Hz, 3H), 7.63 (t, *J* = 7.5 Hz, 3H).

MS (MALDI-TOF, DCTB): m/z = 791.9 $[M]^+$. HRMS (MALDI-TOF, DCTB + PPGNa 790): m/z calculated for $C_{24}H_9BFI_3N_6$: 791.8099; found: 791.8108 (***o*-3a**), 791.8097 (***o*-3b**).

UV-vis (chloroform): λ_{max} (nm) (log ε (dm^3 mol^{-1} cm^{-1})) = 572 (4.90), 534 (sh), 276 (4.60).

FT-IR (ATR): ν (cm^{-1}) = 3062, 2678, 2562, 2443, 1602, 1545, 1451, 1427, 1394, 1312, 1253, 1189, 1167, 1135, 1097, 1076, 1047, 1022, 925, 815, 792, 747, 705, 641, 591.

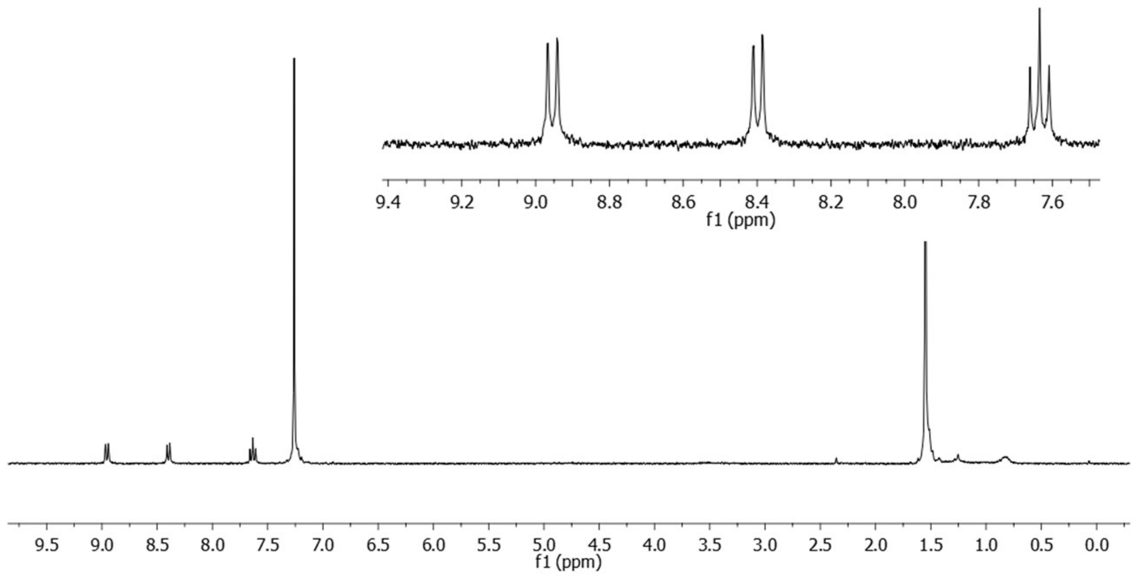

Figure S33. ^{1}H NMR spectrum (300 MHz, chloroform-d_1) of ***o*-3a**.

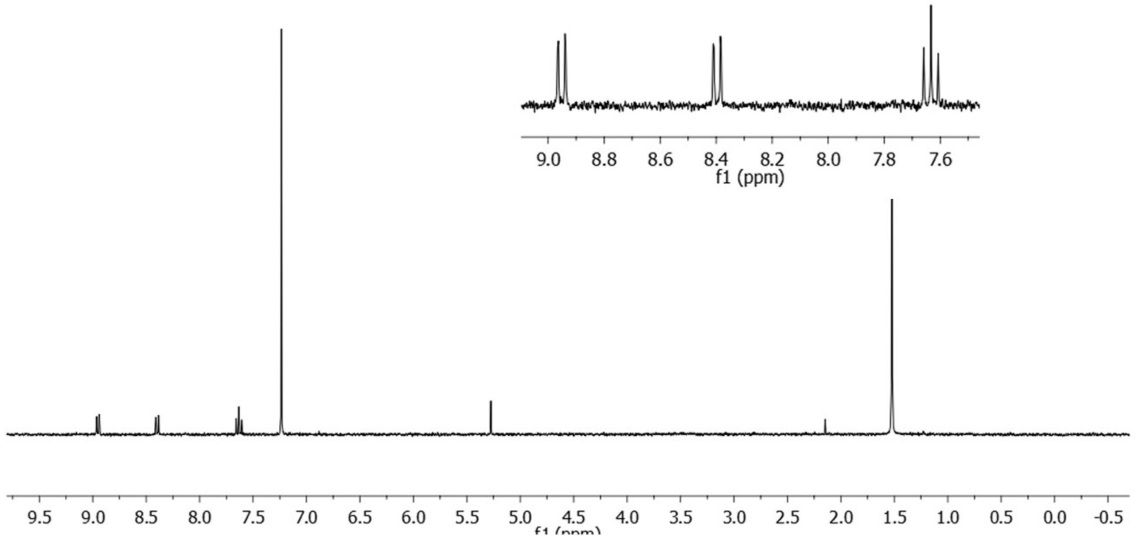

Figure S34. ^{1}H NMR spectrum (300 MHz, chloroform-d_1) of ***o*-3b**.

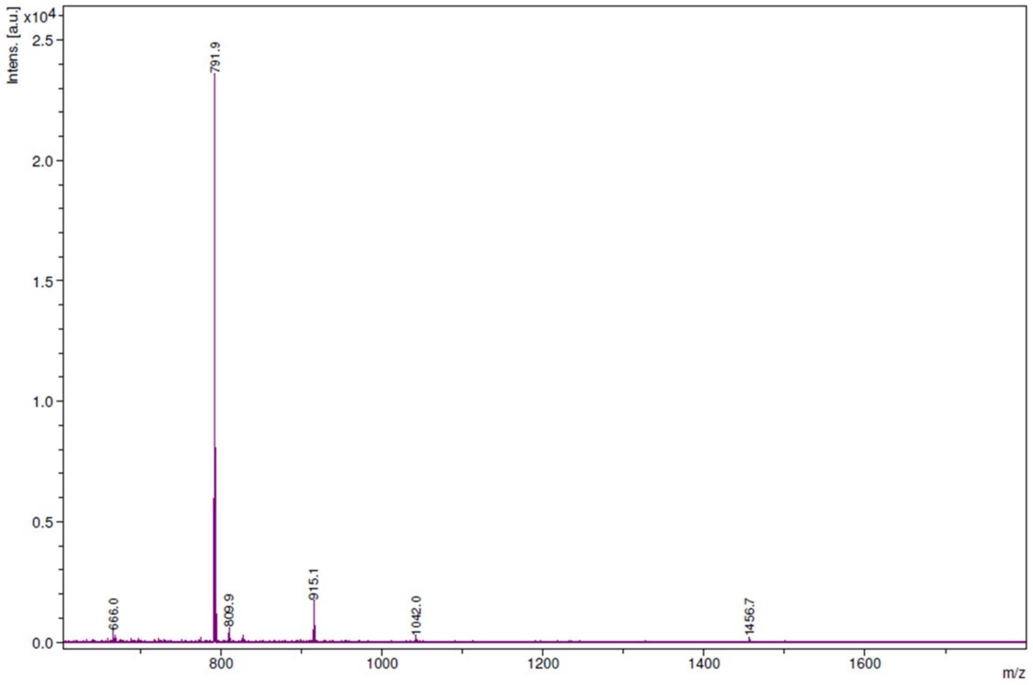

Figure S35. MALDI-TOF mass spectrum (matrix: DCTB) of ***o*-3a**.

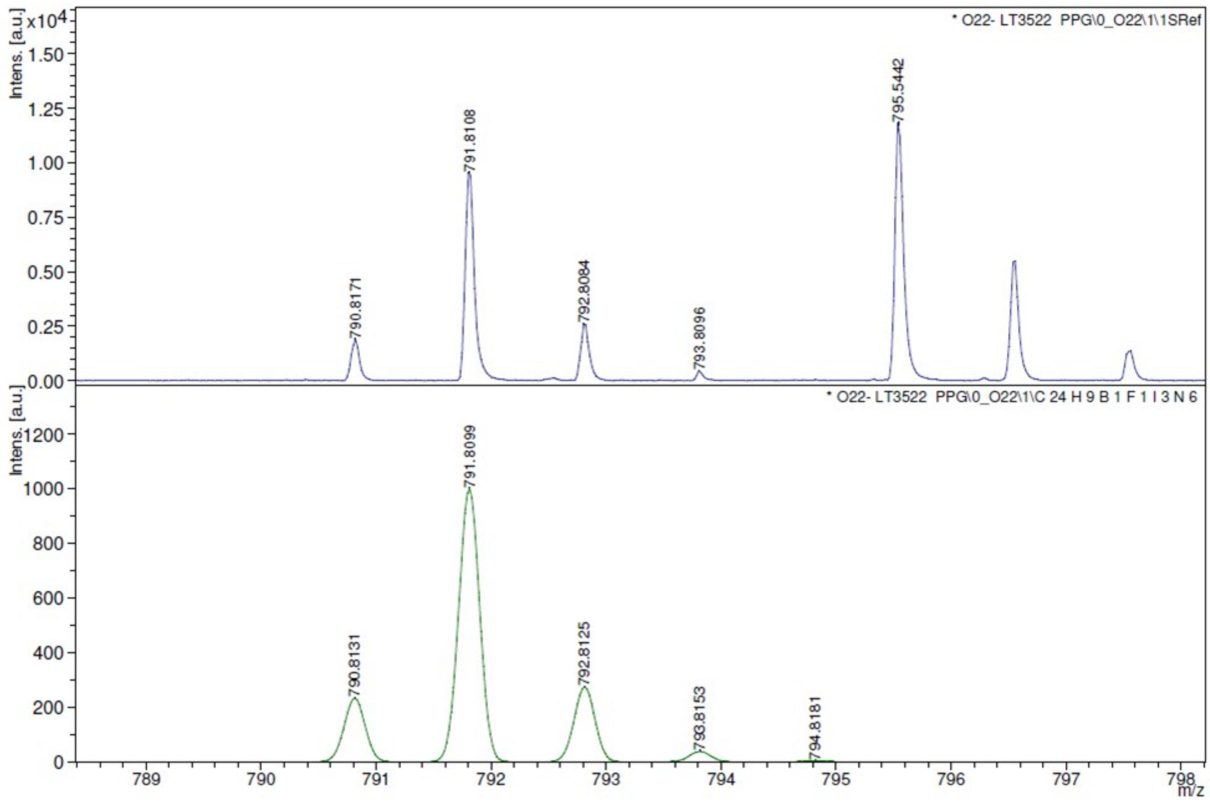

Figure S36. (Top) HR-MS (MALDI-TOF) spectrum (matrix: DCTB + DCTB + PPGNa 790) and (bottom) calculated isotopic pattern of ***o*-3a.**

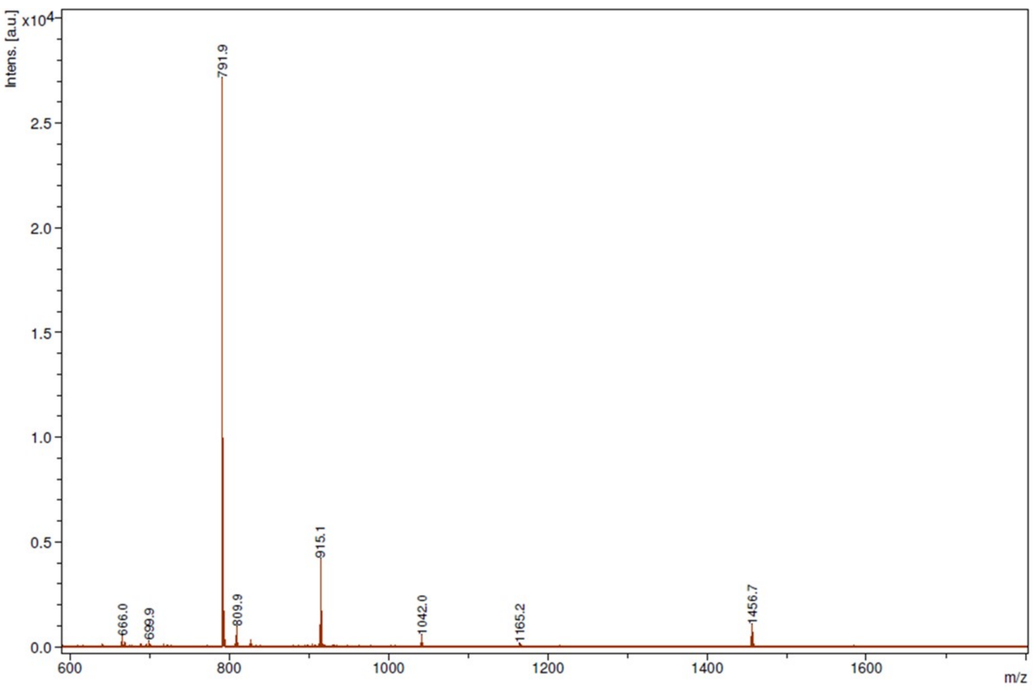

Figure S37. MALDI-TOF mass spectrum (matrix: DCTB) of ***o*-3b**.

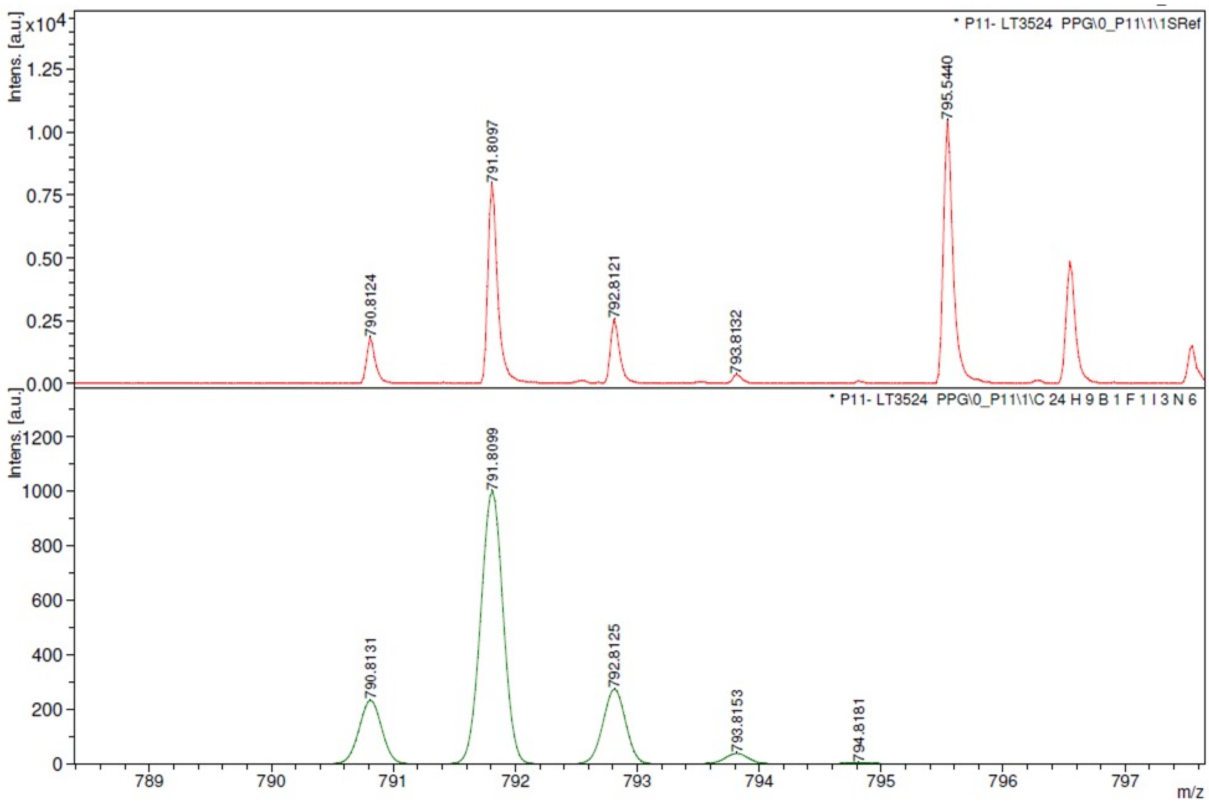

Figure S38. (Top) HR-MS (MALDI-TOF) spectrum (matrix: DCTB + DCTB + PPGNa 790) and (bottom) calculated isotopic pattern of ***o*-3b**

4. Analysis of inherent chirality of C_3-symmetric SubPcs

Due to their cone-shaped geometry of the SubPc macrocycle, SubPcs are intrinsically non-centrosymmetric. Thus, each of the C_1-and C_3-symmetric SubPc regioisomers obtained from cyclotrimerization reaction of a non-C_{2v}-symmetric phthalonitrile precursor (namely, *meta*- or *ortho*-monosubstituted phthalonitriles) is inherently chiral and is obtained as a racemic mixture of enantiomers (*i.e.*, *P* and *M* for the C_3 regioisomer, and *MPP* and *MMP* for the C_1 regioisomer).

In order to assign the absolute configuration to the enantiomers of C_3-symmetric SubPcs, the following convention is followed, which makes use of the *P*/*M* stereodescriptors system commonly employed to designate the chirality of buckybowl molecules and exploits the CIP sequence rules (Figure S39).[3,4] 1) The SubPc is placed having the axial ligand pointing towards the observer. 2) The rim atoms that rank highest in priority according to the CIP rules (which for trisubstituted, C_3-SubPcs coincide with the carbon atoms bearing the peripheral substituent giving rise to the C_3-symmetric substitution pattern) are identified and considered as points of origin. 3) For each point of origin, the two rim atoms attached to this point are compared, and the same is done, if necessary, for subsequent atoms attached thereto, until two rim atoms that lie the same number of positions away from the point of origin on the clockwise and counterclockwise paths are found that differ in CIP priority. 4) If the direction of travel from the point of origin to the rim atom with higher CIP priority is clockwise, the SubPc is designated as *P*; if the direction of travel is anticlockwise, the SubPc is designated as *M*.

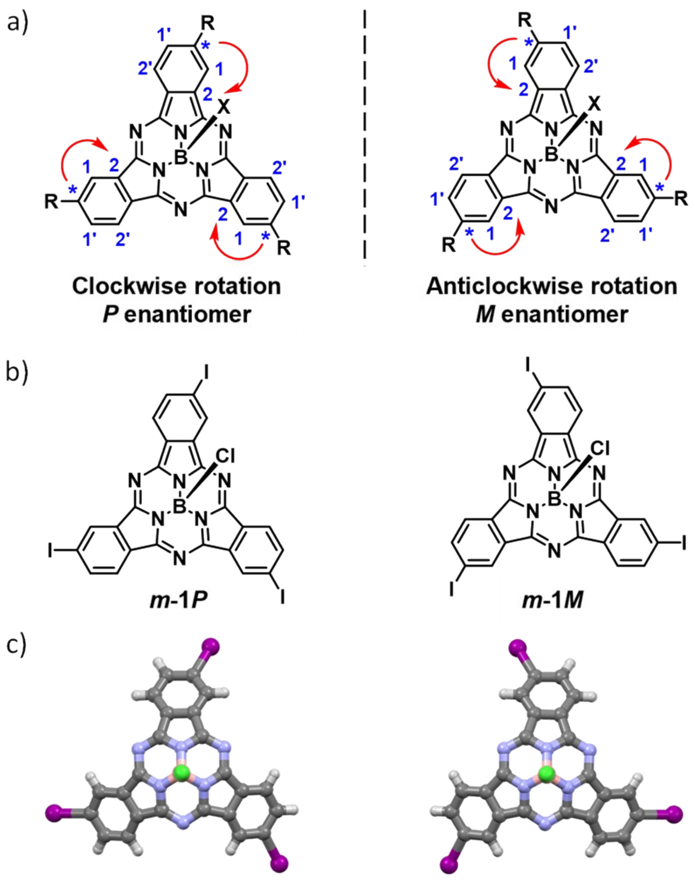

Figure S39. a) General chemical structure of a C_3-symmetric, *meta*-substituted SubPc and convention for the assignment of the *P* or *M* configuration. The rim atoms that rank highest in priority (points of origin) are marked with an asterisk. Whereas 1 and 1' have the same priority according to the CIP rules, 2 ranks higher in priority with respect to 2'. b) Chemical structure and c) molecular modelling of the (left) *P* and (right) *M* enantiomers of C_3-symmetric I_3SubPc-Cl ***m*-1**.

5. Additional experimental data

5.1 X-ray diffraction analysis

*X-ray diffraction analysis of **o-2*** (first eluted fraction)*

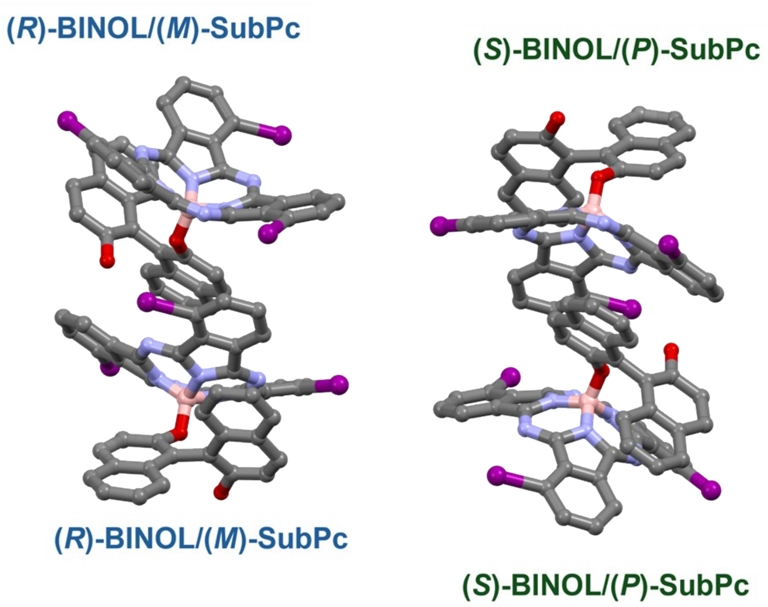

Figure S40. View of the crystal structure of the first eluted fraction obtained from column chromatography on silica gel of the product obtained by reacting ***o*-1** with (*R*)-BINOL in refluxing chlorobenzene in the presence of DBU as a base (***o*-2***), showing the presence of both enantiomers of the BINOL moiety species as a result of the racemization of the chiral auxiliary (Deposition number CCDC 2376453). The crystal structure contains solvent molecules with different degree of disorder in the interstices, which have been omitted for clarity.

Table S1. Sample and crystal data for the first eluted fraction of the product obtained by reacting ***o*-1** with (*R*)-BINOL in refluxing chlorobenzene in the presence of DBU as a base (***o*-2***, deposition number CCDC 2376453).

**Chemical formula**	$C_{45}H_{24.5}BCl_3I_3N_6O_{3.75}$	
**Formula weight**	1207.06 g/mol	
**Temperature**	296.15 K	
**Wavelength**	0.71073 Å	
**Crystal size**	0.002 x 0.013 x 0.039 mm	
**Crystal habit**	dark purple needle	
**Crystal system**	Triclinic	
**Space group**	P -1	
**Unit cell dimensions**	a = 14.1186(9) Å	α = 80.404(3)°
	b = 14.7801(8) Å	β = 75.708(3)°
	c = 24.1650(14) Å	γ = 75.885(3)°
**Volume**	4708.5(5) $Å^3$	
**Z**	4	
**Density (calculated)**	1.703 g cm^{-3}	
**Absorption coefficient**	2.209 mm^{-1}	
**F(000)**	2326.0	

Table S2. Data collection and structure refinement for the first eluted fraction of the product obtained by reacting ***o*-1** with (*R*)-BINOL in refluxing chlorobenzene in the presence of DBU as a base ((***o*-2***, deposition number CCDC 2376453).

**2Θ range for data collection**	3.046 to 50.7°	
**Index ranges**	-17 ≤h ≤17, -17 ≤k ≤17, -29 ≤l ≤29	
**Reflections collected**	111890	
**Independent reflections**	17186 [R_{int} = 0.0689, R_{sigma} = 0.0593]	
**Coverage of independent reflections**	99.9%	
**Absorption correction**	multi-scan	
**Structure solution technique**	direct methods	
**Structure solution program**	XT, VERSION 2018/2	
**Refinement method**	Full-matrix least-squares on F^2	
**Refinement program**	SHELXL-2019/1 (Sheldrick, 2019)	
**Function minimized**	$\Sigma w(F_o^2 - F_c^2)^2$	
**Data / restraints / parameters**	17186/1044/1105	
**Goodness-of-fit on F^2**	1.038	
**Final R indices**	I>2σ(I)	R_1 = 0.0799, wR_2 = 0.2483
	all data	R_1 = 0.1449, wR_2 = 0.3081
**Weighting scheme**	$w=1/[\sigma^2(F_o^2)+(0.2000P)^2]$	
	where $P=(F_o^2+2F_c^2)/3$	
**Largest diff. peak/hole**	3.37/-1.22 $eÅ^{-3}$	

X-ray diffraction analysis of ***m-3***

A clear intense purple ribbon-like specimen of $C_{51}H_{28}BBr_2I_3N_6O_2$, approximate dimensions 0.026 mm x 0.038 mm x 0.323 mm, was used for the X-ray crystallographic analysis. The X-ray intensity data were measured.

The total exposure time was 89.56 hours. The frames were integrated with the Bruker SAINT software package using a narrow-frame algorithm. The integration of the data using a triclinic unit cell yielded a total of 35582 reflections to a maximum θ angle of 25.35° (0.83 Å resolution), of which 16119 were independent (average redundancy 2.207, completeness = 99.9%, R_{int} = 8.62%, R_{sig} = 15.97%) and 9274 (57.53%) were greater than $2\sigma(F^2)$. The final cell constants of a = 9.5889(10) Å, b = 14.2119(15) Å, c = 17.0045(15) Å, α = 94.584(6)°, β = 102.778(6)°, γ = 91.388(6)°, volume = 2250.6(4) $Å^3$, are based upon the refinement of the XYZ-centroids of 3151 reflections above 20 σ(I) with 5.12° < 2θ < 45.65°. Data were corrected for absorption effects using the multi-scan method (SADABS). The ratio of minimum to maximum apparent transmission was 0.799. The calculated minimum and maximum transmission coefficients (based on crystal size) are 0.3650 and 0.9050.

The structure was solved and refined using the Bruker SHELXTL Software Package, using the space group P 1, with Z = 2 for the formula unit, $C_{51}H_{28}BBr_2I_3N_6O_2$. The final anisotropic full-matrix least-squares refinement on F^2 with 425 variables converged at R1 = 9.81%, for the observed data and wR2 = 23.90% for all data. The goodness-of-fit was 1.046. The largest peak in the final difference electron density synthesis was 3.245 $e^-/Å^3$ and the largest hole was -4.994 $e^-/Å^3$ with an RMS deviation of 0.256 $e^-/Å^3$. On the basis of the final model, the calculated density was 1.930 g/cm^3 and F(000), 1252 e^-.

Table S3. Sample and crystal data for ***m*-3** (mixture of diastereomers, deposition number CCDC 2376454).

**Chemical formula**	$C_{51}H_{28}BBr_2I_3N_6O_2$	
**Formula weight**	1308.12 g/mol	
**Temperature**	200(2) K	
**Wavelength**	0.71073 Å	
**Crystal size**	0.026 x 0.038 x 0.323 mm	
**Crystal habit**	clear intense purple ribbon	
**Crystal system**	Triclinic	
**Space group**	P 1	
**Unit cell dimensions**	a = 9.5889(10) Å	α = 94.584(6) °
	b = 14.2119(15) Å	β = 102.778(6)°
	c = 17.0045(15) Å	γ = 91.388(6)°
**Volume**	2250.6(4) $Å^3$	
**Z**	2	
**Density (calculated)**	1.930 g cm^{-3}	
**Absorption coefficient**	3.909 mm^{-1}	
**F(000)**	1252	

Table S4. Data collection and structure refinement for ***m*-3** (mixture of diastereomers, deposition number CCDC 2376454).

**Theta range for data collection**	1.23 to 25.35°	
**Index ranges**	-11<=h<=11, -17<=k<=17, -20<=l<=20	
**Reflections collected**	35582	
**Independent reflections**	16119 [R(int) = 0.0862]	
**Coverage of independent reflections**	99.9%	
**Absorption correction**	multi-scan	
**Max. and min. transmission**	0.9050 and 0.3650	
**Structure solution technique**	direct methods	
**Structure solution program**	SHELXS-97 (Sheldrick 2008)	
**Refinement method**	Full-matrix least-squares on F^2	
**Refinement program**	SHELXL-2014/7 (Sheldrick, 2014)	
**Function minimized**	Σ w(F_o^2 - F_c^2)^2	
**Data / restraints / parameters**	16119 / 3 / 425	
**Goodness-of-fit on F^2**	1.046	
**Final R indices**	9274 data; I>2σ(I)	R1 = 0.0981, wR2 = 0.1982
	all data	R1 = 0.1778, wR2 = 0.2390
**Weighting scheme**	w=1/[σ^2(F_o^2)+(0.0493P)^2+74.6695P]	
	where P=(F_o^2+2F_c^2)/3	
**Absolute structure parameter**	0.0(0)	
**Largest diff. peak and hole**	3.245 and -4.994 eÅ^{-3}	
**R.M.S. deviation from mean**	0.256 eÅ^{-3}	

5.2 HPLC chromatograms

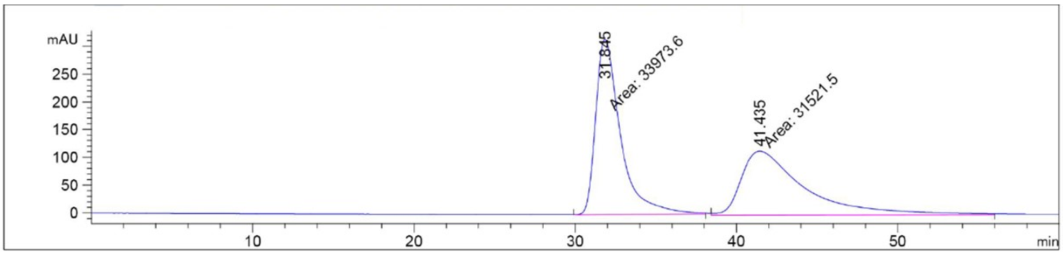

Peak #	RetTime [min]	Type	Width [min]	Area [mAU*s]	Height [mAU]	Area %
1	31.845	MM	1.7946	3.39736e4	315.51517	51.8720
2	41.435	FM	4.5699	3.15215e4	114.95994	48.1280

Figure S41. HPLC chromatogram of ***m*-3** purified as a mixture of diastereomers (see section 2 for further details). Eluting solvent: toluene/*n*-hexane 70:30 (*v*/*v*); flow rate: 1.0 mL min^{-1}; temperature: 10 °C; detection wavelength: 580 nm.

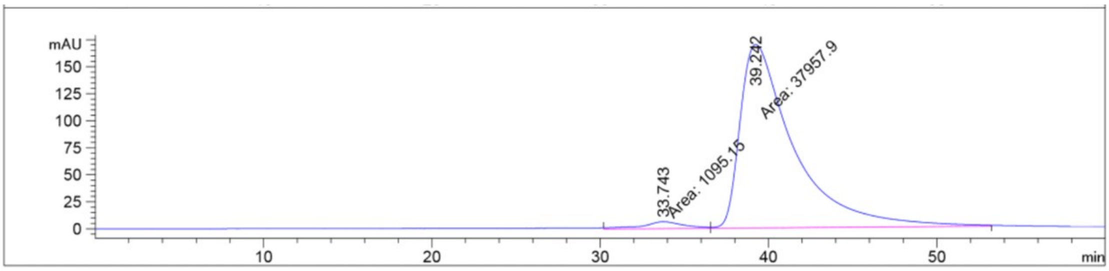

Peak #	RetTime [min]	Type	Width [min]	Area [mAU*s]	Height [mAU]	Area %
1	33.743	MM	2.8748	1095.15369	6.34912	2.8043
2	39.242	MM	3.7238	3.79579e4	169.88863	97.1957

Figure S42. HPLC chromatograms of the isolated ***m*-3a** diastereomer collected as the first eluted fraction from the first column chromatography of ***m*-3** (see section 2 for further details). Eluting solvent: toluene/*n*-hexane 70:30 (*v*/*v*); flow rate: 1.0 mL min^{-1}; temperature: 10 °C; detection wavelength = 580 nm).

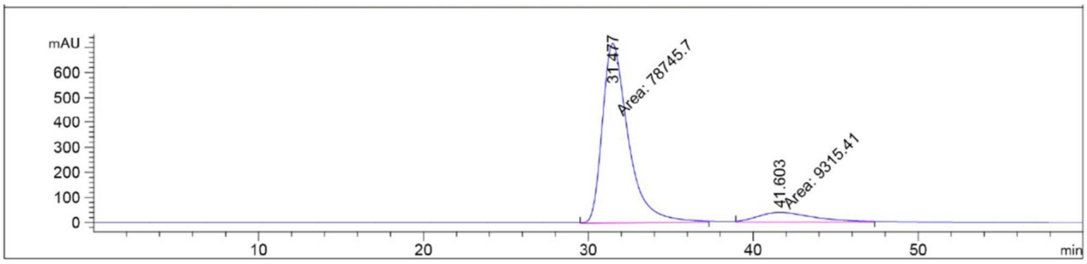

Peak #	RetTime [min]	Type	Width [min]	Area [mAU*s]	Height [mAU]	Area %
1	31.477	MM	1.8271	7.87457e4	718.32257	89.4217
2	41.603	MM	4.0256	9315.40625	38.56778	10.5783

Figure S43. HPLC chromatograms of the isolated ***m*-3b** diastereomer collected as the second eluted fraction from the first column chromatography of ***m*-3** (see section 2 for further details). Eluting solvent: toluene/*n*-hexane 70:30 (*v*/*v*); flow rate: 1.0 mL min^{-1}; temperature: 10 °C; detection wavelength = 580 nm).

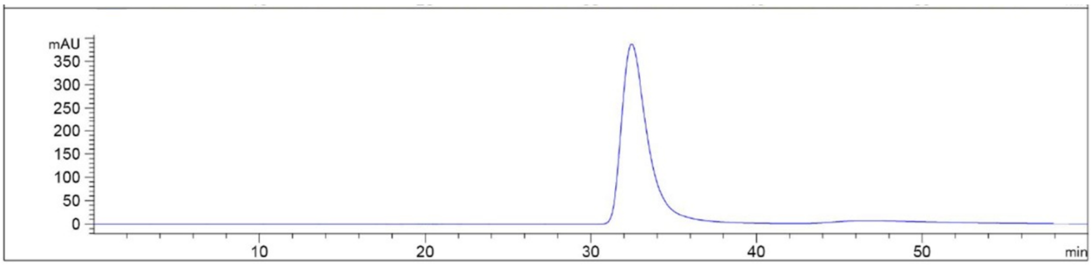

Figure S44. HPLC chromatograms of the isolated ***m*-3b** diastereomer collected as the second eluted fraction from the second column chromatography of ***m*-3** (see section 2 for further details). Eluting solvent: toluene/*n*-hexane 70:30 (*v*/*v*); flow rate: 1.0 mL min^{-1}; temperature: 10 °C; detection wavelength = 580 nm).

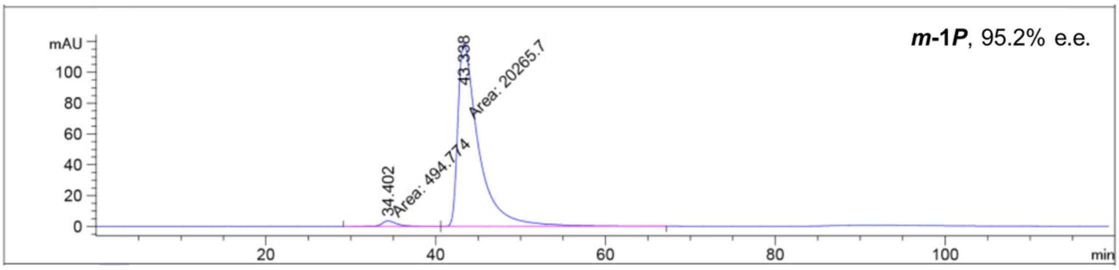

Peak #	RetTime [min]	Type	Width [min]	Area [mAU*s]	Height [mAU]	Area %
1	34.402	MF	2.2746	494.77390	3.62538	2.3833
2	43.338	FM	2.8540	2.02657e4	118.34512	97.6167

Figure S45. HPLC chromatogram of the product obtained from the removal of the chiral auxiliary from ***m*-3a** in the presence of BCl_3 (***m*-1*P***, 95.2% e.e.). Eluting solvent: toluene/*n*-hexane 50:50 (*v*/*v*); flow rate = 1.2 mL min^{-1}; temperature = 10 °C; detection wavelength = 570 nm).

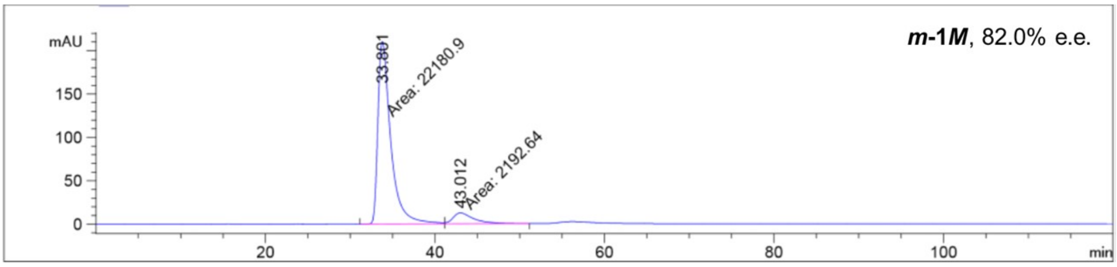

Peak #	RetTime [min]	Type	Width [min]	Area [mAU*s]	Height [mAU]	Area %
1	33.801	MF	1.7587	2.21809e4	210.19666	91.0040
2	43.012	FM	2.9015	2192.63550	12.59478	8.9960

Figure S46. HPLC chromatogram of the product obtained from the removal of the chiral auxiliary from ***m*-3b** in the presence of BCl_3 (***m*-1*M***, 82.0% e.e.). Eluting solvent: toluene/*n*-hexane 50:50 (*v*/*v*); flow rate = 1.2 mL min^{-1}; temperature = 10 °C; detection wavelength = 570 nm).

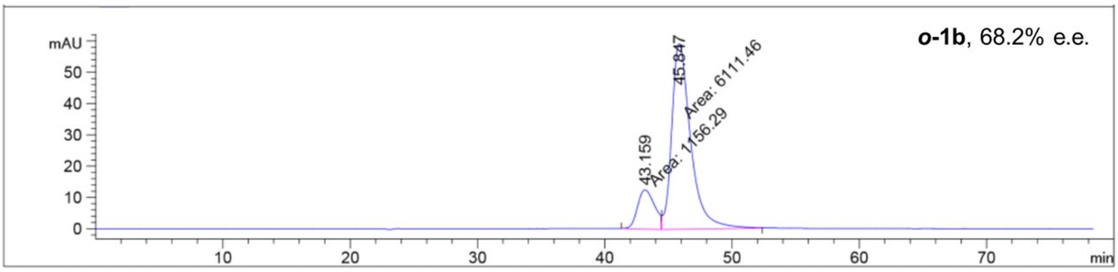

Peak #	RetTime [min]	Type	Width [min]	Area [mAU*s]	Height [mAU]	Area %
1	43.159	MM	1.5367	1156.28589	12.54109	15.9098
2	45.847	MM	1.7189	6111.46240	59.25865	84.0902

Figure S47. HPLC chromatogram of the product obtained from the removal of the chiral auxiliary from ***o*-2b** in the presence of BCl_3 (***o*-1b**, 68.2% e.e.). Eluting solvent: toluene/*n*-hexane 50:50 (*v*/*v*); flow rate = 0.8 mL min^{-1}; temperature = 10 °C; detection wavelength = 580 nm).

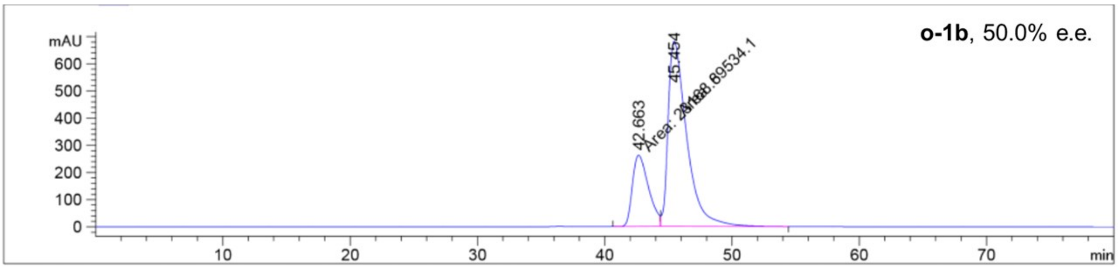

Peak #	RetTime [min]	Type	Width [min]	Area [mAU*s]	Height [mAU]	Area %
1	42.663	MM	1.4726	2.31688e4	262.22064	24.9925
2	45.454	MM	1.7016	6.95341e4	681.08447	75.0075

Figure S48. HPLC chromatogram of the product obtained by further treatment of the ***o*-1b** fraction obtained from the removal of the chiral auxiliary from ***o*-2b** in the presence of BCl_3 (68.2% e.e.) with BCl_3 for 1h (***o*-1b**, 50.0% e.e.). Eluting solvent: toluene/*n*-hexane 50:50 (*v*/*v*); flow rate = 0.8 mL min^{-1}; temperature = 10 °C; detection wavelength = 580 nm).

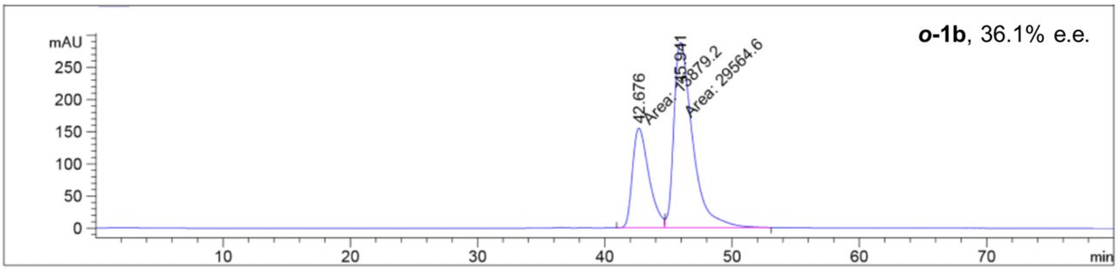

Peak #	RetTime [min]	Type	Width [min]	Area [mAU*s]	Height [mAU]	Area %
1	42.676	MM	1.4964	1.38792e4	154.58003	31.9475
2	45.941	MM	1.7126	2.95646e4	287.71381	68.0525

Figure S49. HPLC chromatogram of the product obtained by further treatment of the ***o*-1b** fraction obtained from the removal of the chiral auxiliary from ***o*-2b** in the presence of BCl_3 (68.2% e.e.) with BCl_3 for 2h (***o*-1b**, 36.1% e.e.). Eluting solvent: toluene/*n*-hexane 50:50 (*v*/*v*); flow rate = 0.8 mL min^{-1}; temperature = 10 °C; detection wavelength = 580 nm).

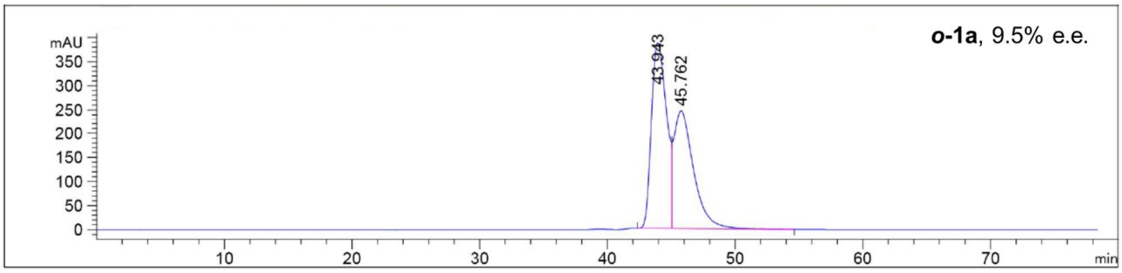

Peak #	RetTime [min]	Type	Width [min]	Area [mAU*s]	Height [mAU]	Area %
1	43.943	BV	1.2857	3.18148e4	385.93518	54.7350
2	45.762	VB	1.5710	2.63104e4	245.76056	45.2650

Figure 50. HPLC chromatogram of the product obtained from the removal of the chiral auxiliary from ***o*-2a** in the presence of BCl_3 (***o*-1a**, 9.5% e.e.). Eluting solvent: toluene/*n*-hexane 50:50 (*v*/*v*); flow rate = 0.8 mL min^{-1}; temperature = 10 °C; detection wavelength = 580 nm).

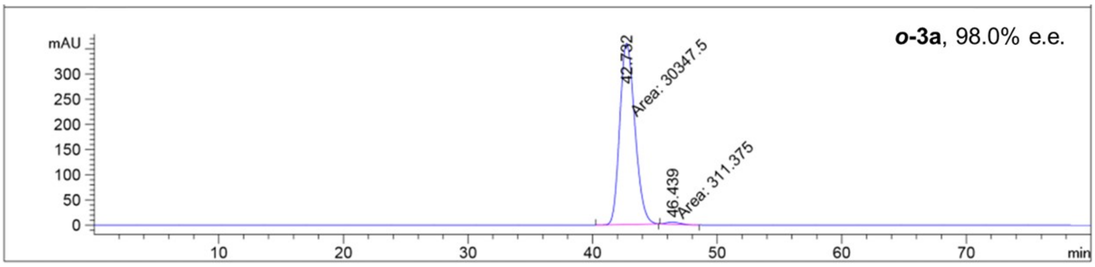

Peak #	RetTime [min]	Type	Width [min]	Area [mAU*s]	Height [mAU]	Area %
1	42.732	MM	1.4072	3.03475e4	359.42770	98.9844
2	46.439	MM	1.2907	311.37497	4.02088	1.0156

Figure S51. HPLC chromatogram of the product obtained from the removal of the chiral auxiliary from ***o*-2a** in the presence of BF_3·Et_2O (***o*-3a**, 98.0% e.e.). Eluting solvent: toluene/*n*-hexane 80:20 (*v*/*v*); flow rate = 0.6 mL min^{-1}; temperature = 20 °C; detection wavelength = 580 nm).

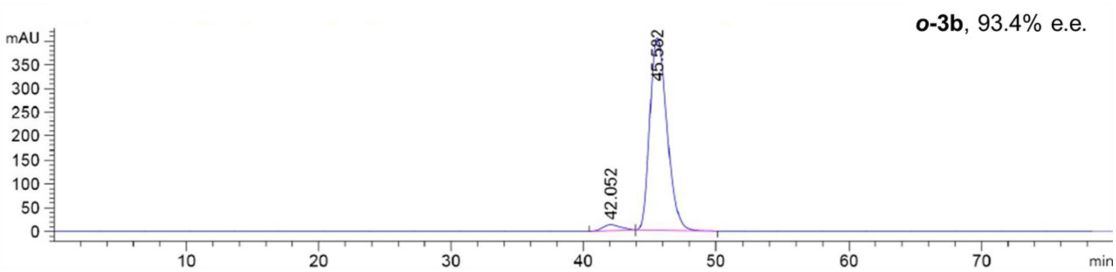

Peak #	RetTime [min]	Type	Width [min]	Area [mAU*s]	Height [mAU]	Area %
1	42.052	BB	1.2356	1242.18115	13.17958	3.2980
2	45.582	BB	1.4097	3.64226e4	404.03259	96.7020

Figure S52. HPLC chromatogram of the product obtained from the removal of the chiral auxiliary from ***o*-2b** in the presence of BF_3·Et_2O (***o*-3b**, 93.4% e.e.). Eluting solvent: toluene/*n*-hexane 80:20 (*v*/*v*); flow rate = 0.6 mL min^{-1}; temperature = 20 °C; detection wavelength = 580 nm).

5.3 Circular dichroism and UV-vis absorption spectra

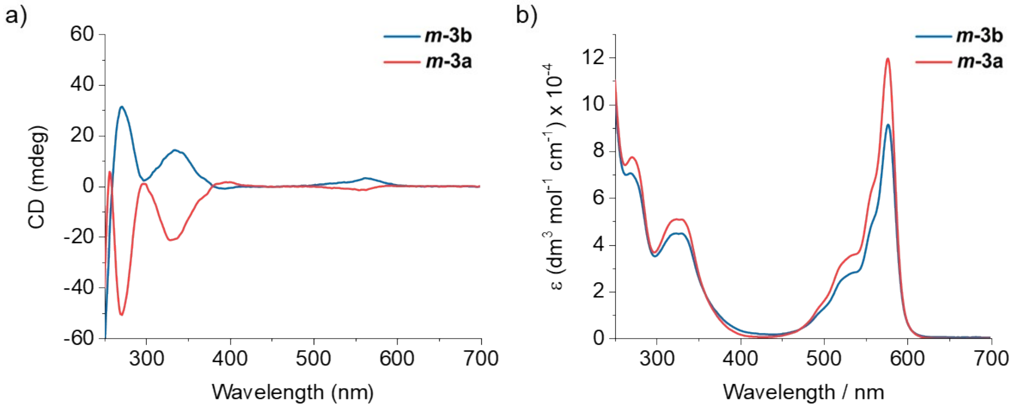

Figure S53. a) CD spectra of ***m*-3a** (red spectrum) and ***m*-3b** (blue spectrum) in $CHCl_3$ (2.0×10^{-5} M). b) UV-vis absorption spectra of ***m*-3a** (red spectrum) and ***m*-3b** (blue spectrum) in $CHCl_3$ (7.0×10^{-6} M).

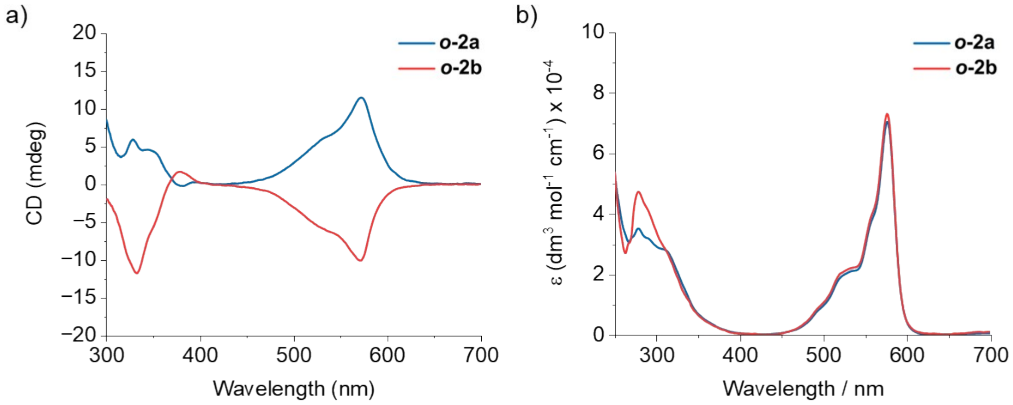

Figure S54. a) CD spectra of ***o*-2a** (blue spectrum) and ***o*-2b** (red spectrum) in $CHCl_3$ (5.0×10^{-5} M). b) UV-vis absorption spectra of ***o*-2a** (blue spectrum) and ***o*-2b** (red spectrum) in $CHCl_3$ (5.0×10^{-5} M).

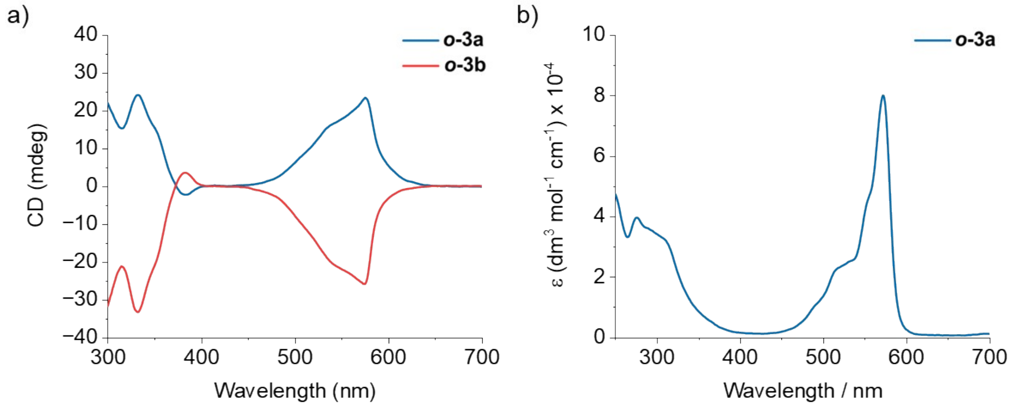

Figure S55. a) CD spectra of ***o*-3a** (blue spectrum) and ***o*-3b** (red spectrum) in $CHCl_3$ (5.0×10^{-5} M). b) UV-vis absorption spectra of ***o*-2a** in $CHCl_3$ (5.0×10^{-5} M).

6. Author contributions

T. T. and M. V. M.-D. conceived the project, supervised the research and acquired funding. G.L. conducted the experiments related to the *meta*-substituted derivatives and L.T. realized the experiments on the *ortho*-substituted ones. G.L. wrote the manuscript. All authors discussed the results and revised the manuscript.

7. References

[1] I. Sanchez-Molina, B. Grimm, R. M. Krick Calderon, C. G. Claessens, D. M. Guldi, T. Torres, *J. Am. Chem. Soc.* **2013**, *135*, 10503.

[2] C. G. Claessens, T. Torres, *Eur. J. Org. Chem.* **2000**, 1603-1607.

[3] M. A. Petrukhina, K. W. Andreini, L. Peng, L. T. Scott, *Angew. Chem. Int. Ed.* **2004**, *43*, 5477-5481.

[4] K. Kanagaraj, K. Lin, W. Wu, G. Gao, Z. Zhong, D. Su, C. Yang, *Symmetry* **2017**, *9*, 174.
